# Temperature and Geographic Location Impact the Distribution and Diversity of Photoautotrophic Gene Variants in Alkaline Yellowstone Hot Springs

**DOI:** 10.1128/spectrum.01465-21

**Published:** 2022-05-16

**Authors:** Annastacia C. Bennett, Senthil K. Murugapiran, Eric D. Kees, Hailey M. Sauer, Trinity L. Hamilton

**Affiliations:** a Department of Plant and Microbial Biology, University of Minnesota, St. Paul, Minnesota, USA; b Biotechnology Institute, University of Minnesota, St. Paul, Minnesota, USA; State Key Laboratory of Microbial Resources, Institute of Microbiology, Chinese Academy of Sciences

**Keywords:** anoxygenic photosynthesis, Chloroflexi, cyanobacteria, hot springs, metagenomics, photosynthesis, phototroph

## Abstract

Alkaline hot springs in Yellowstone National Park (YNP) provide a framework to study the relationship between photoautotrophs and temperature. Previous work has focused on studying how cyanobacteria (oxygenic phototrophs) vary with temperature, sulfide, and pH, but many questions remain regarding the ecophysiology of anoxygenic photosynthesis due to the taxonomic and metabolic diversity of these taxa. To this end, we examined the distribution of genes involved in phototrophy, carbon fixation, and nitrogen fixation in eight alkaline (pH 7.3-9.4) hot spring sites near the upper temperature limit of photosynthesis (71ºC) in YNP using metagenome sequencing. Based on genes encoding key reaction center proteins, geographic isolation plays a larger role than temperature in selecting for distinct phototrophic Chloroflexi, while genes typically associated with autotrophy in anoxygenic phototrophs, did not have distinct distributions with temperature. Additionally, we recovered Calvin cycle gene variants associated with Chloroflexi, an alternative carbon fixation pathway in anoxygenic photoautotrophs. Lastly, we recovered several abundant nitrogen fixation gene sequences associated with Roseiflexus, providing further evidence that genes involved in nitrogen fixation in Chloroflexi are more common than previously assumed. Together, our results add to the body of work on the distribution and functional potential of phototrophic bacteria in Yellowstone National Park hot springs and support the hypothesis that a combination of abiotic and biotic factors impact the distribution of phototrophic bacteria in hot springs. Future studies of isolates and metagenome assembled genomes (MAGs) from these data and others will further our understanding of the ecology and evolution of hot spring anoxygenic phototrophs.

**IMPORTANCE** Photosynthetic bacteria in hot springs are of great importance to both microbial evolution and ecology. While a large body of work has focused on oxygenic photosynthesis in cyanobacteria in Mushroom and Octopus Springs in Yellowstone National Park, many questions remain regarding the metabolic potential and ecology of hot spring anoxygenic phototrophs. Anoxygenic phototrophs are metabolically and taxonomically diverse, and further investigations into their physiology will lead to a deeper understanding of microbial evolution and ecology of these taxa. Here, we have quantified the distribution of key genes involved in carbon and nitrogen metabolism in both oxygenic and anoxygenic phototrophs. Our results suggest that temperature >68ºC selects for distinct groups of cyanobacteria and that carbon fixation pathways associated with these taxa are likely subject to the same selective pressure. Additionally, our data suggest that phototrophic Chloroflexi genes and carbon fixation genes are largely influenced by local conditions as evidenced by our gene variant analysis. Lastly, we recovered several genes associated with potentially novel phototrophic Chloroflexi. Together, our results add to the body of work on hot springs in Yellowstone National Park and set the stage for future work on metagenome assembled genomes.

## INTRODUCTION

Decades of research in Yellowstone National Park (YNP) hot springs show chlorophototrophic (chlorophyll-dependent phototrophs, herein “phototrophs”) bacteria exhibit a temperature-dependent distribution, wherein eukaryotic algae predominate in acidic hot springs at <56ºC, and phototrophic cyanobacteria and Chloroflexi prevail in alkaline hot springs at >60ºC (1–5). In alkaline environments, temperature can exhibit further control over the distribution of a given cyanobacterial genus. This is most evident in the distribution of *Synechococcus*, wherein *Synechococcus* ecotypes are partitioned by 1ºC increments within mats in Mushroom and Octopus Springs (6). Beyond cyanobacteria, anoxygenic phototrophs also exhibit variable distributions with temperature in Octopus and Mushroom Springs (5, 7, 8) and in a handful of additional alkaline springs in YNP revealed by single-marker gene surveys (9–14). However, anoxygenic phototrophs are a metabolically and taxonomically diverse group with few characterized hot springs isolates, and broad distributions of these taxa in YNP hot springs are not well understood. Here, we aim to explore the idea that geographic isolation and temperature play important roles in environmental and geographic selection of anoxygenic phototrophs, an ongoing debate noted in the Becraft et al. (2011) study of cyanobacterial ecotypes (6).

Isolate studies and *in situ* experiments provide important insight into genetic content and physiology within a given environment and are crucial to fully determine the role of specific taxa in an ecosystem (2). However, the lack of isolate genomes of high temperature, alkaline hot spring, anoxygenic phototrophs limits our understanding of their physiology. While there are at least 90 alkaline hot spring cyanobacteria genomes available (15), there are only eight alkaline hot spring Chloroflexi isolate genomes to date (Roseiflexus castenholzii HL08, *Roseiflexus* sp. R2-1, Roseiflexus sp. R2-2, Chloroflexus aggregans DSM 9485, Chloroflexus aurantiacus Y-400-fl, Chloroflexus aurantiacus OK-70-fl, Chloroflexus aurantiacus J-10-fl, and Chloroflexus islandicus isl-2).

Chloroflexi are the most abundant and widespread anoxygenic phototroph in alkaline hot springs (4, 5, 10, 11, 16, 17). Phototrophy (photoautotrophy, photomixotrophy, and photoheterotrophy) in phylum Chloroflexi is limited to class Chloroflexales with one exception, “Candidatus Roseilinea” (18, 19). Unlike cyanobacteria that rely on the Calvin cycle for autotrophy, photoautotrophic Chloroflexi (meaning both photoautotrophs and photomixotrophs) genomes can vary in carbon assimilation genes: both Chloroflexus and Roseiflexus genomes contain genes for the autotrophic 3-hydroxypropionate bicycle (3-HPB) (20, 21), but only Chloroflexus isolates have been grown in the absence of acetate (22, 23). Herein, we refer to carbon-fixing Chloroflexi as photoautotrophs, but acknowledge that they can live a photomixotrophic lifestyle or chemoautotrophic lifestyle, dependent on light conditions, time of day, or presence of suitable electron donors (21, 23, 24). To tease apart carbon assimilation by both taxa *in situ*, Klatt et al. (2013) assessed expression of key genes in the 3-hydroxypropionate pathway (3-HPB) at 60ºC and 65ºC in Mushroom Spring. In that study, transcripts of 3-HPB in Roseiflexus were observed at 65ºC and Chloroflexus at 60ºC, which suggests taxon-specific temperature partitioning of these genera (8). Here, we expand this work by surveying both anoxygenic photosynthesis reaction center genes and key carbon fixation genes across high temperature gradients (62ºC to 71ºC) to determine if this pattern occurs across a broader range of hot springs in YNP.

Alkaline hot springs in YNP are limited in nitrogen, which selects for nitrogen-fixing bacteria, diazotrophs (14, 25). Nitrogen fixation is catalyzed by the enzyme nitrogenase, which is energetically and metabolically expensive (26). Nitrogenase is an iron-sulfur complex containing one of three metals harbored in the active site: molybdenum (Mo), iron (Fe), or vanadium (V). Mo-nitrogenase is the most common and is encoded by nif genes (27, 28). Several studies have assessed potential nitrogenase activity in acidic hot springs of >55ºC using the gene *nifH*, which encodes the iron protein (NifH) in nitrogenase (29–31). These studies suggested diazotrophs in acidic hot springs are adapted to local conditions. In alkaline hot spring mats (53–63ºC), the abundance of Synechococcus *nifH* transcripts increased in the evening once mats turn anoxic (8, 32). Roseiflexus genomes (including hot spring isolates) contain *nif* genes, but they lack the full protein suite required to build a functional nitrogenase and likely do not fix nitrogen. However, Roseiflexus *nifH* transcripts have been observed at 57ºC and 68ºC in Mound Spring in YNP, suggesting a functional purpose that remains unknown (e.g., reference 33).

Given the abundance of cyanobacteria and Chloroflexi in alkaline hot springs and the crucial role they play in nitrogen and carbon cycling, we sought to determine the role of temperature in constraining the distribution of key genes for photosynthesis and nitrogen fixation in eight alkaline hot springs with temperatures ranging from 62–71ºC. We examined genes and pathways associated with phototrophy, autotrophy, and nitrogen fixation in metagenome assemblies, an approach that has been informative in other systems (e.g., reference 34). We found that (i) genes associated with photosynthetic machinery are abundant throughout our samples and richness is lower in 62ºC sites, (ii) operational taxonomic units (OTUs) of taxa commonly associated with alkaline hot springs (Synechococcus, Roseiflexus, and Chloroflexus) as well as novel Chloroflexi OTUs (Roseilinea and “unclassified” Chloroflexi) are present in our samples, (iii) RuBisCO gene variant distribution suggests adaption to local conditions, and (iv) 3HPB genes are abundant throughout our samples. In addition, we recovered several NifH protein sequences related to Roseiflexus, a taxon that could be important for discerning the evolutionary history of nitrogen fixation. In general, our OTU analysis suggest taxa are largely influenced by local conditions. Temperatures of >68ºC select for distinct groups of cyanobacteria, while geographic location selects for phototrophic Chloroflexi and carbon fixation genes. These results add to the body of work on photoautotrophic bacteria in alkaline hot springs, which is critical to solving the evolutionary history and ecophysiology of nitrogen fixation and photosynthesis in bacteria.

## RESULTS AND DISCUSSION

### Overview of site geochemistry and study design.

**FIG 1:**
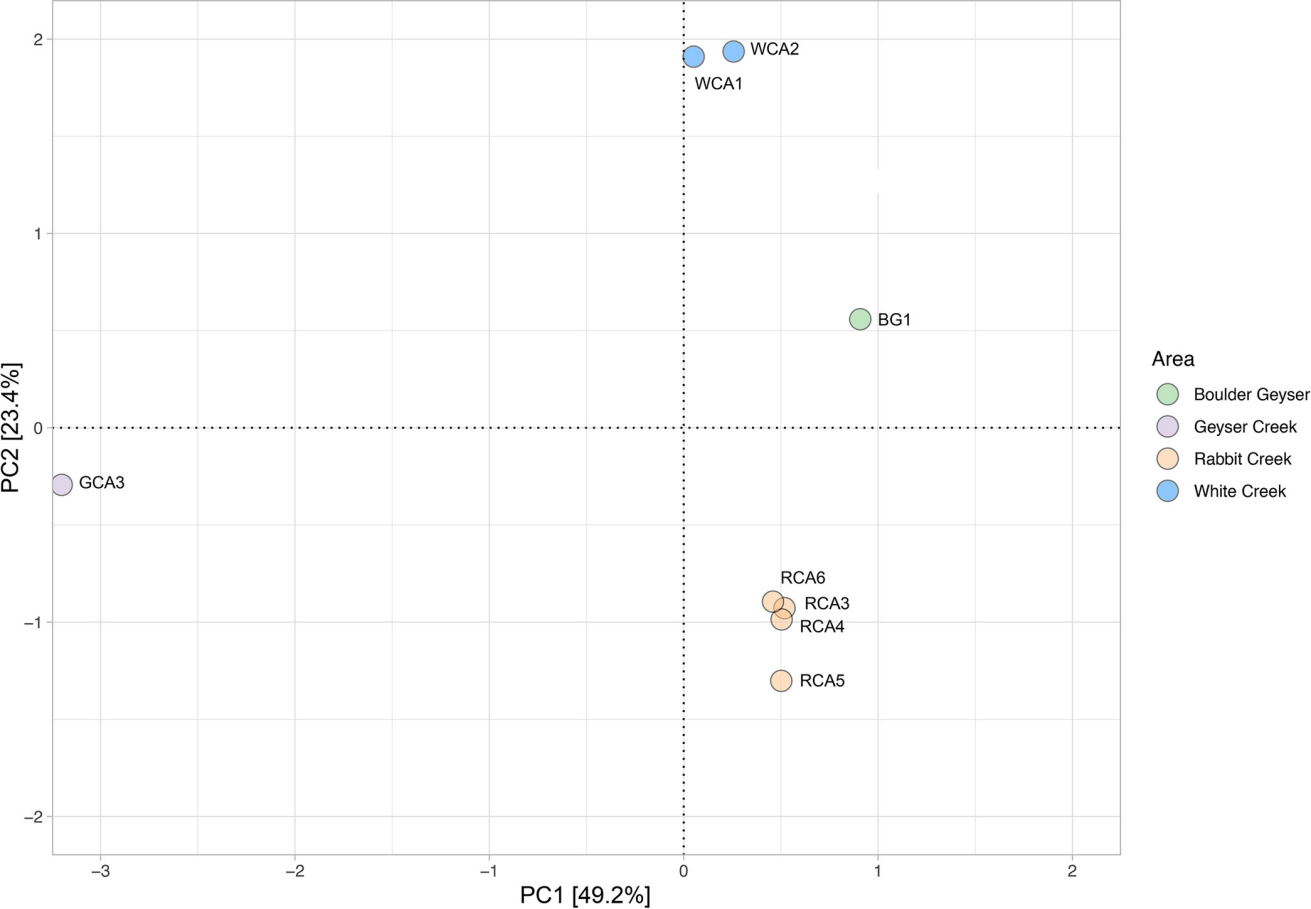
Principal component analysis of site meta-data. Principal components were calculated using the numeric data in Table S1A. Sites are labeled by site ID in corresponding Table S1 and shaded by Yellowstone National Park area.

16S rRNA gene sequencing has been conducted in several alkaline hot springs in YNP and has been useful in determining putative phototrophic taxa (reviewed in reference 4). Based on previous 16S rRNA amplicon sequencing, we found that putative phototrophs, including Synechococcus, Roseiflexus, and Chloroflexus, were abundant in eight different hot spring sites in YNP. These sites ranged in temperature from 62ºC to 71ºC, pH between 7 and 9, and sample morphology included mats, pinnacles, and filaments (Table S1 in the supplemental material) (11). In general, the sites cluster by geothermal area while temperature, dissolved organic carbon, sulfide, and iron were also major drivers of dissimilarity (Fig. 1). Here, we leveraged metagenome sequencing to determine ecological diversity and metabolic potential of phototrophic bacteria in eight alkaline springs that have not been the focus of historic work in YNP. While Mushroom and Octopus springs are alkaline, they differ compared to our sites in terms of morphology, geochemistry, and geographic location. We generated metagenomes to determine the diversity, distribution, and abundance of specific genes involved in phototrophy, autotrophy, and nitrogen fixation. Because diversity at the 16S rRNA gene level decreases with increasing temperature and geographic isolation plays a role in structuring hot spring communities (6, 8, 35, 36), we hypothesized that these factors would also impact the distribution, diversity, and abundance of functional genes. To this end, we calculated Shannon diversity for each target gene in our eight hot spring sites and examined gene abundance by mapping metagenome reads to genes of interest in the assembled metagenomes.

### Geographic isolation plays a role in the diversity and distribution of cyanobacterial photosystem genes.

**FIG 2:**
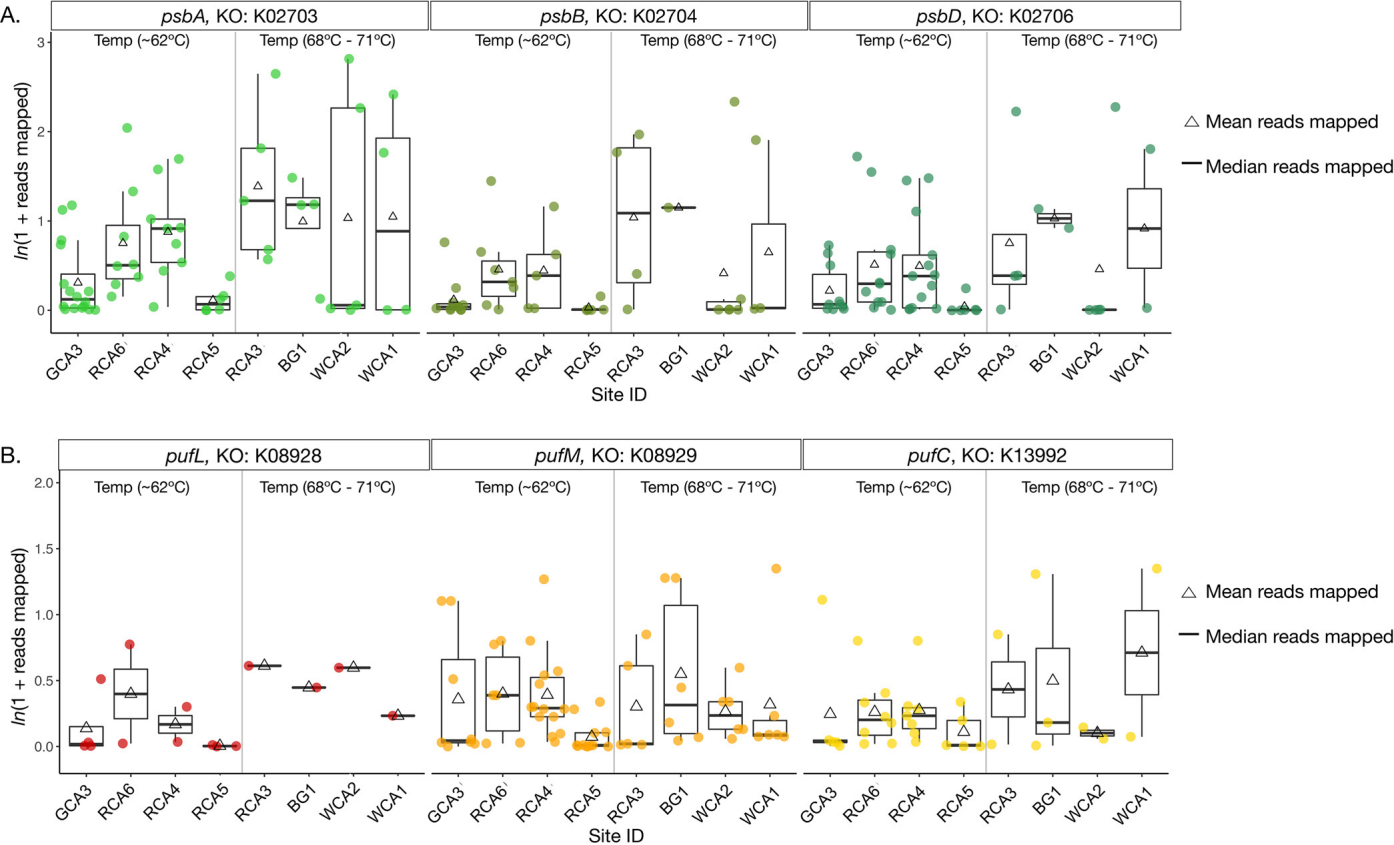
Distribution of photosynthetic genes with temperature. The overall abundance (normalized ln(1 + reads mapped)) of genes that encode for Cyanobacterial photosystem II (*psb*) and type II anoxygenic photosynthesis reaction centers (*puf*) are shown as box plots for each site. Triangles represent the mean abundance for the gene set, and dots represent individual gene abundances, shaded and separated by the corresponding photosystem or reaction center gene (KEGG Orthology IDs are shown with gene name). Boxes represent the inter quartile range (Q1–Q3) and whiskers (lines) represent the maximum and minimum, with outliers removed (±2.5 standard deviations from the mean). A gray line divides the sites into high temperature and low temperature groups. Sites are ordered by increasing temperature.

Oxygenic photosynthesis is a remarkable metabolism that involves two photosystems, Photosystem I (PSI) and Photosystem II (PSII), working in concert to harvest electrons from water to fuel carbon fixation and other cellular processes. PSII houses the oxygen-evolving complex and antenna proteins where light energy is captured to liberate electrons from water—a process that requires expression of several proteins that are encoded by psb genes (37–39). We quantified the abundance of three key *psb* genes: *psbA, psbB*, and *psbD* (Fig. 2A). The *psbA* and *psbD* genes encode for the D1 and D2 proteins, respectively, which both serve to ligate the redox-active components in PSII and are highly transcriptionally regulated in cyanobacteria (37). The *psbB* gene encodes CP47, a chlorophyll binding protein crucial to forming a stable PSII reaction center; taxa with multiple copies of *psbB* are acclimated to far-red light (40). While we observed a range of sequence abundances from rare (0.001) to 3 normalized reads mapped, we did not observe statistically significant differences in photosystem gene abundance in our data (Table S2) nor a decrease in abundance with increasing temperature (Fig. 2A).

**FIG 3:**
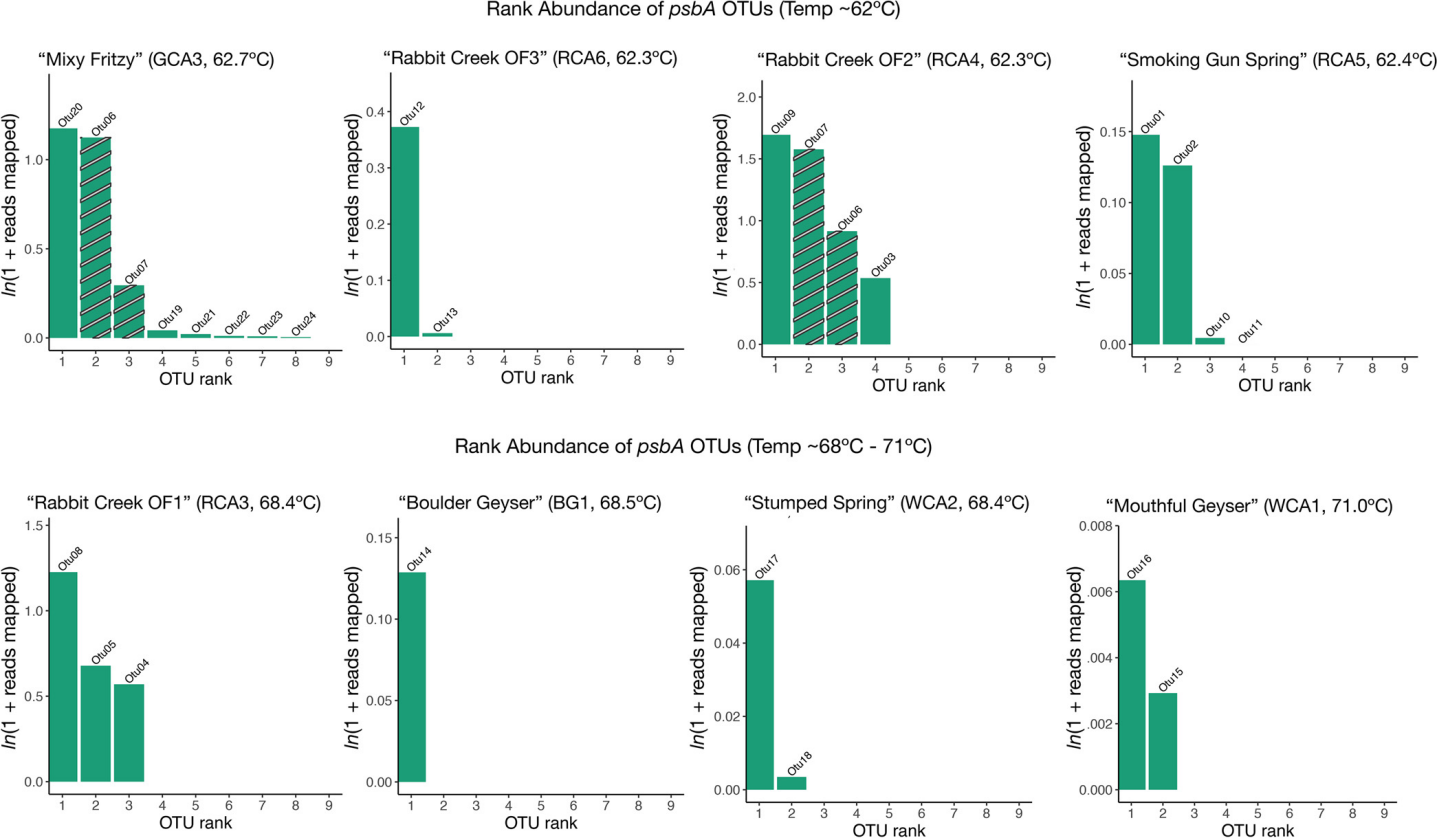
Richness and distribution of *psbA* gene variants. Rank abundance plots for each site are displayed in increasing temperature order. Plots display abundances as normalized ln(1 + reads mapped) for each *psbA* OTU, and OTUs are ranked in order from most to least abundant. Bars are labeled with the OTU number. Striped bars represent OTUs that are present in more than one site.

We classified *psbA* genes into operational taxonomic units (OTUs, 99% nucleotide similarity, reference database in supplemental material) resulting in 27 *psbA* OTUs (Fig. 3, Figure S1). To examine diversity in taxonomy of the psbA OTUs, we translated *psbA* to PsbA and ran both phylogenetic and BLASTP analyses (41) (Fig. S1). Based on the phylogenetic placement of PsbA and our BLASTP results, 14 of 24 OTUs were classified as Synechococcus. Several OTUs were related to the high temperature strains JA-2-3B'a (2–13) and 63AY4M2. Two PsbA OTUs, OTU15 and OTU17, were most closely related to strain 63AY4M2 and were present in our highest temperature sites, WCA1 (71.0ºC) and WCA2 (69.4ºC) (Fig. S1). Both reference strains were isolated from Mushroom and Octopus Springs, where temperatures range from 60–65ºC (42), and our results may suggest a range for strain 63AY4M2 beyond 65ºC. While strain-level distribution cannot be discerned from these data alone, future work should be done to determine the genomic variation in Synechococcus strains beyond Mushroom and Octopus Springs (see reference 43). While the majority of the OTUs recovered here were *Synechococcus*, we also recovered OTUs that were most closely related to *Gloeomargarita lithophora*, *Thermosynechococcus* sp., and *Leptolyngbya* sp. in 62ºC sites. These observations are consistent with previous work suggesting cyanobacterial diversity increases with decreasing temperature in alkaline hot springs (7, 9, 10).

While the abundance of photosynthesis reaction center genes did not correlate with temperature, we did observe differences in photosystem gene copy number. For example, in our highest temperature sites (68–71ºC), there were few highly abundant *psbA* sequences while at lower temperatures there were more less abundant *psbA* sequences. We expected that the > 68ºC samples would contain distinct *psbA* variants compared to the lower temperature sites because temperature selects for ecotypes that vary in photosynthetic properties in Mushroom and Octopus Springs (3, 5–7). We found that *psbA* variants were largely site specific (Fig. 3) and alpha diversity across sites did not correlate with temperature (Fig. S2A), highlighting that geographic isolation could play a selective role in this environment.

In general, *psbA* richness was higher in 62ºC sites compared to others (Fig. 3). In the 62ºC sites, only two OTUs were present in more than one site (OTU06 and OTU07 in site RCA4 and site GCA3), and in the high temperature sites, all *psbA* OTUs were unique. Both OTUs were associated with *Synechococcus* OH strains capable of growth up to 70ºC in pure culture (44). We observed several abundant OTUs in Rabbit Creek sites (RCA, sites RCA3, RCA4, and RCA6), where our previous 16S rRNA analysis revealed abundant *Synechococcus* 16S rRNA gene sequences (11). The recovery of multiple psbA OTUs in each RCA site is consistent with the presence of multiple *Synechococcus* strains or ecotypes with several distinct copies of *psbA*. Fewer distinct OTUs in sites of  >63ºC is consistent with strain (or ecotype) adaption at higher temperatures, like what was observed in Octopus spring (7).

### Chloroflexi photosystem genes have distinct distributions with temperature and reveal novel taxa.

Given that cyanobacteria photosystem genes did not follow a distinct temperature pattern, but PsbA OTU analysis revealed gene variants are largely site specific, we sought to determine if anoxygenic phototrophs followed a similar pattern. Anoxygenic phototrophs commonly observed at temperatures of  >60ºC have type-II reaction centers that are encoded by *puf* genes (45), and the majority of anoxygenic phototrophs in hot springs of >60ºC are phototrophic Chloroflexi (7, 11, 20). Here we surveyed puf genes to examine if the diversity of putative phototrophic Chloroflexi (class Chloroflexales and *Candidatus* Thermofonsia) also decreases with increasing temperature (Fig. 2B). *pufL* and *pufM* encode PufL and PufM, membrane-spanning proteins that bind bacteriochlorophylls in type-II reaction centers, while *pufC* gene encodes a cytochrome involved in photosynthetic electron transfer (19, 45, 46). *puf* gene abundances ranged from rare (0.001 normalized reads mapped) to 1.5 normalized reads mapped (Fig. 2B). We recovered more copies of *pufLC* genes in sites of <68ºC, which is consistent with a decrease in genetic (or taxonomic) diversity with increasing temperature, as seen in Mushroom Spring, Octopus Spring, and Rabbit Creek (3, 5, 10–12, 17). In contrast, several copies of *pufM* genes were abundant in all sites. Together, these results suggest that taxa with type-II reaction centers could encode multiple copies of *pufM*. Furthermore, our data suggest that diversity of anoxygenic phototrophs decreases with increasing temperature or taxa at temperatures 62ºC contain multiple copies of *pufLC*. The presence of multiple copies of *puf* genes has not been confirmed in Chloroflexi isolate genomes, but in other phyla gene homologs are necessary for adaption to changing environmental conditions (47) and should be investigated further in phototrophic Chloroflexi.

**FIG 4:**
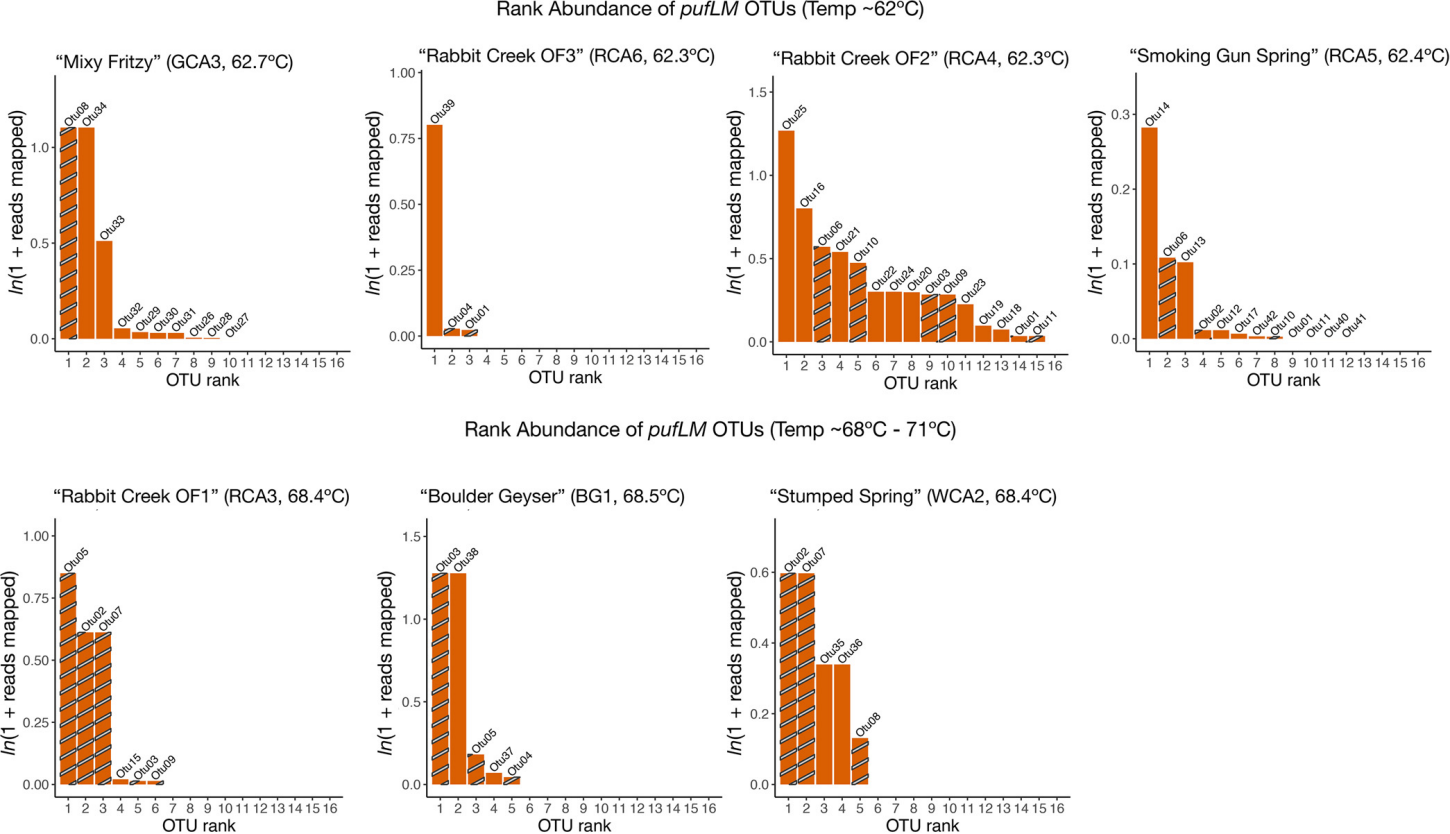
Richness and distribution of *pufLM* gene variants. Rank abundance plots for each site are displayed in increasing temperature order. Plots display abundances as normalized ln(1 + reads mapped) for each *pufLM* OTU, and OTUs are ranked in order from most to least abundant. Bars are labeled with the OTU number. Striped bars represent OTUs that are present in more than one site.

To determine the diversity of *puf* genes in these sites, we assigned OTUs to our concatenated and translated *pufLM* genes (at 99% similarity) and assigned taxonomy using BLASTP (41). We found that *pufLM* diversity did not correlate with temperature (Fig. S2B). We recovered 42 *pufLM* OTUs across seven sites (Fig. 4, Fig, S3). Thirty-five of the 42 OTUs were affiliated with Chloroflexi. Of the seven non-Chloroflexi OTUs, none were in the top 20 most abundant OTUs; five were Proteobacteria and two were Actinobacteria. Previous work has shown that phototrophic Proteobacteria are rare in alkaline hot springs at >60ºC (4, 9, 10), and non-Chloroflexi pufLM OTUs were not abundant in our metagenomes. We found that our most abundant and most common OTUs were Roseiflexus (OTU05) and Chloroflexus (OTU03) genera (Fig. S3), which is consistent with both our previous 16S rRNA gene analysis (10, 11), and 16S rRNA and metatranscriptomic analyses in Mushroom and Octopus Springs (5, 7, 20, 48).

The present metagenomic sequencing data set provides higher resolution than our previous 16S rRNA gene analysis (11). Our metagenomic sequencing approach resulted in the recovery of taxa that have not been identified in YNP hot springs at present. Three of our top 20 most abundant OTUs were assigned “*Candidatus* Roseilinea sp. NK_OTU-006” by BLASTP. The only described species from this class is “*Candidatus* Roseilinea sp. strain NK_OTU-006,” recovered from sulfidic hot springs in Japan near 56ºC (18). Our *Ca*. Roseilinea-like *pufLM* OTUs (OTU23, 24, and 33) were found in two alkaline sites low in sulfide (RCA4 and GCA3), both with temperatures of 62ºC, pushing the geographic range and upper temperature limit of this novel class. Furthermore, eight of our *pufLM* OTUs were assigned “Chloroflexi bacterium” by BLASTP (Table in Fig. S3B), suggesting novel Chloroflexi are present in these hot spring sites.

In Mushroom Spring, Klatt et al. (2013), observed *Roseiflexus* in 60ºC and *Chloroflexus* transcripts in 65ºC sites, indicating temperature partitioning of the two phototrophic Chloroflexi genera (8). Our data are consistent with the Mushroom Spring study but suggest temperature partitioning of the two genera at higher temperatures: we recovered putative Roseiflexus OTUs in sites up to 68ºC and putative *Chloroflexus* OTUs in sites up to 69ºC. We also observed more *Chloroflexus* than *Roseiflexus* OTUs in 68ºC–71ºC sites (Fig. S3C). Recovery of cyanobacterial *psb* genes and Chloroflexi *puf* genes from the same sites is consistent with several historical studies postulating the presence of “green non-sulfur bacteria” co-occurring with cyanobacteria in Mushroom and Octopus spring mats (49–53). Recent works have examined the distribution of phototrophic Chloroflexi using single marker genes (9–14), and our data support the hypothesis that both phototrophic taxa persist at temperatures of >68ºC with two different optimal temperatures: *Roseiflexus* up to 68ºC and *Chloroflexus* up to 69ºC. Future work is needed to determine if this hypothesis holds true with *Roseiflexus* and *Chloroflexus* metagenome assembled genomes or hot spring isolates.

### Calvin cycle genes have distinct distributions with temperature while 3HPB genes are widespread and abundant.

Photoautotrophic bacteria fix the majority of carbon in alkaline geothermal springs using the Calvin-Benson-Bassham (Calvin) cycle (cyanobacteria, some Chloroflexi), the reductive tricarboxylic acid (rTCA) cycle (class Chlorobia), or the 3-hydroxypropionate bicycle (3HPB, most photoautotrophic Chloroflexi) (reviewed in reference 54). Recent work has shed light on the flexibility of carbon fixation in Chloroflexi in high temperature, alkaline hot springs: *Roseiflexus* and *Chloroflexus* in Mushroom and Octopus springs contain genes for the 3HPB, but a handful of studies have recovered Calvin cycle genes in phototrophic “*Candidatus* Thermofonsia” (55) and “*Candidatus* Chlorohelix allophototropha” (56), and nonphototrophic class Anaerolineaea (57). The carboxylation step in the Calvin cycle is carried out by the enzyme ribulose 1,5 bisphosphate carboxylase/oxygenase: RuBisCO (encoded by *rbcL* [large subunit] and *rbcS* [small subunit] genes). In hot springs specifically, *Synechococcus* species have evolved a thermotolerant form of RuBisCO that can function up to 74ºC (58). Phosphoribulokinase (encoded by the *prk* gene), a second essential step of the Calvin cycle, does not appear to have an upper temperature limit beyond that of phototrophy, but is likely only present in organisms that use the Calvin cycle (59).

**FIG 5:**
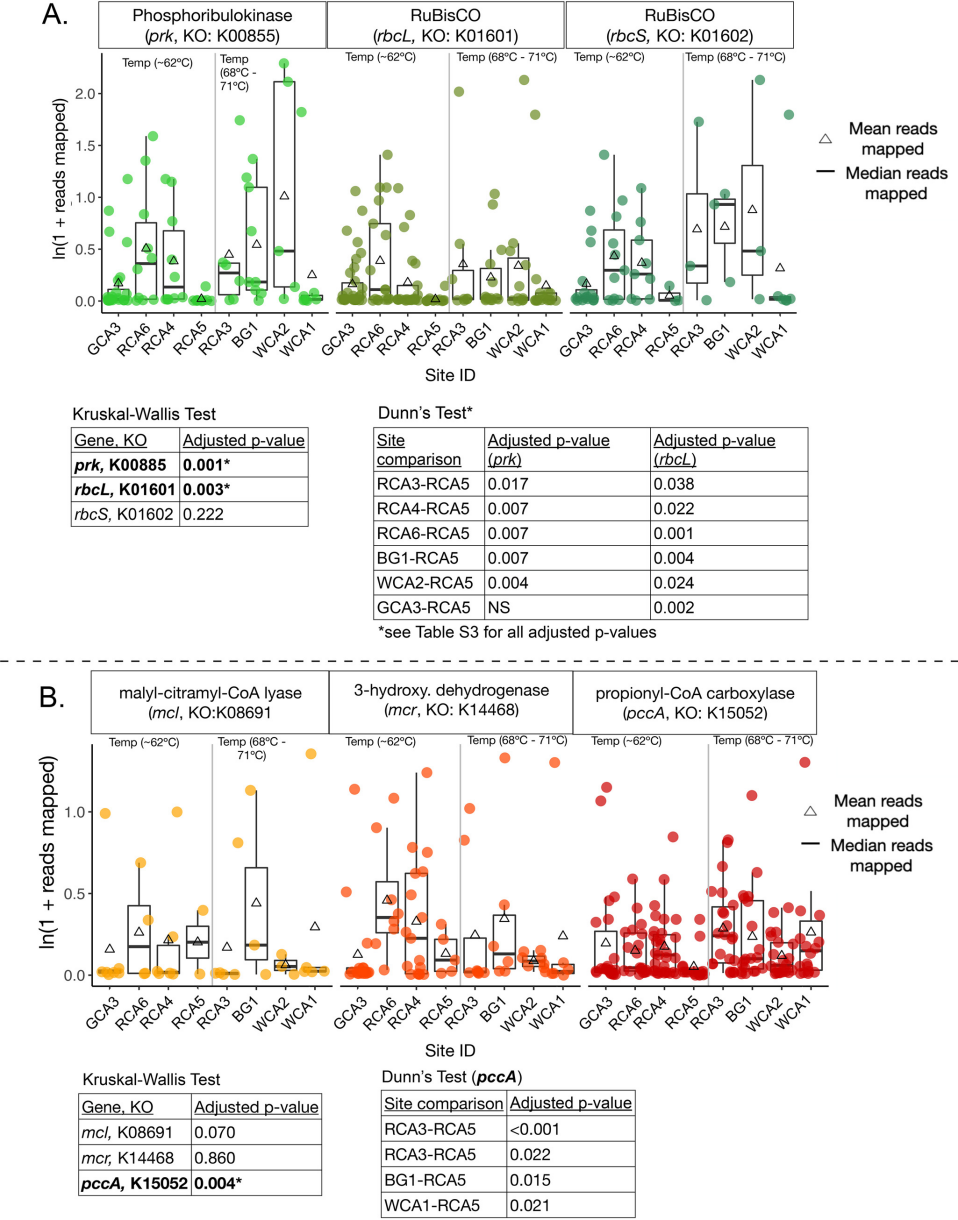
Abundance and distribution of key genes in phototrophic carbon fixation pathways. The abundance (normalized ln(1 + reads mapped)) of key genes in the Calvin cycle (A) and the 3-hydroxypropionate bicycle (B) are shown as box plots for each site. Triangles represent the mean abundance for the gene set, and dots represent individual gene abundances, shaded by the genes. Boxes represent the inter quartile range (Q1–Q3), and whiskers (lines) represent the maximum and minimum, with outliers removed (±2.5 standard deviations from the mean). Sites are ordered by increasing temperature. A gray line divides the sites into high temperature and low temperature groups. Sites are ordered by increasing temperature. To determine significant differences in gene abundance in all sites, a Kruskal-Wallis H test followed by Dunn’s Multiple Comparison *post hoc* test for significant differences between sites. Only Bonferroni-adjusted *P* values < 0.05 are shown for brevity (all site comparison adjusted *P* values are shown in Table S3).

Given the wide distribution of the genes for the Calvin Cycle in nature (60), we sought to constrain the distribution of *rbcL, rbcS*, and *prk* alkaline hot spring samples and relate these data to our phototroph gene analysis. In contrast to the *psb* analyses, pairwise comparisons of the abundance of both *prk* and *rbcL* showed a statistically significant difference in site RCA5 compared to all other sites, except for the highest temperature site (WCA1) (Fig. 5A). Furthermore, we observed larger mean abundances of *rbcS* than *rbcL*, but more copies of *rbcL* than *rbcS*, suggesting the taxa encoding Calvin cycle genes could encode more copies of *rbcL* or multiple forms of RuBisCO are present in these high temperature, alkaline hot springs. At present, four forms of RuBisCO exist in nature: form I RuBisCO (cyanobacteria, alpha-, beta-, gamma-proteobacteria, Chloroflexi, and autotrophic eukaryotes) contains both the large and small subunits (encoded by *rbcL* and *rbcS* genes, respectively), while forms II (alpha-, beta-, gamma- proteobacteria) and III (only in methanogenic archaea) contain only the large subunit (59, 61, 62). To this end, we calculated the ratio of *rbcL:rbcS* with temperature (Fig. S4). A ratio of 1:1 in *rbcL:rbcS* genes would be indicative of form I RubisCO, while any larger ratio would suggest several form I RuBisCO taxa with extra copies of *rbcL* or the presence of form II and form III taxa. In general, we found ratios of  >1:1 in all sites, with the largest differences in sites at <63ºC. Because more *rbcL* copies are present at lower temperatures, we infer that taxa encoding form II or III RuBisCO (rbcL only, noncyanobacterial Calvin cycle) persist at lower temperatures while form I (cyanobacterial-Calvin cycle) are more prevalent at temperature  >63ºC.

We recovered 77 *rbcL* OTUs (99% nucleotide similarity, reference database in supplemental material) among our eight sites (Fig. S5). We observed fluctuating *rbcL* richness (Fig. S5) and diversity (Fig. S2D) in both sites of  >68ºC and 62ºC sites (Fig. S2D). The majority of our *rbcL* OTUs were site-specific, consistent with adaptation to local conditions and/or geographic isolation. Two exceptions were OTU01 (Armatimonadetes) and OTU02 (*Synechococcus*): OTU01 was present in both high temperature sites and in a 63ºC Rabbit Creek site (RCA4, 62.3ºC), while OTU02 was present in our two highest temperature sites (WCA1, 71ºC; WCA2, 68.4ºC). Given that *rbcL* is commonly associated with cyanobacteria and some Chloroflexi and *psbA* and *rbcL* analyses suggest a combination of local conditions rather than temperature alone is selecting for taxa that encode these two genes, we postulate that these taxa are subject to geographic isolation in alkaline hot springs.

Genes involved in 3HPB, the carbon fixation pathway in most photoautotrophic Chloroflexi, were widespread and abundant in our metagenomes (Fig. 5B). The 3HPB requires two carboxylation steps (via acetyl-CoA carboxylase and propionyl-CoA carboxylase), followed by steps that generate 3-hydroxypropionate and glyoxylate intermediates (54, 63). To this end, we surveyed the abundance of three genes involved in three critical steps in the 3HPB: malyl-CoA/citramyl-CoA lyase (*mcl* gene, glyoxylate generation), propionyl-CoA carboxylase (*pccA* gene, CO2 carboxylation), and 3-hydroxypropionate dehydrogenase (*mcr* gene, 3-hydroxypropionate generation). Only one gene (*pccA*) returned statistically significant differences in abundance across sites. *pccA* abundance was different in site RCA5 (62.5ºC) compared to three high temperature sites (RCA3, BG1, WCA1) and one 62ºC site (RCA4). However, *mcl* and *mcr* in the 3HPB pathway showed no significant difference in abundance across sites. These results are likely because we recovered several low-abundance (<0.01 normalized reads mapped) *pccA* reads in addition to the high abundance reads. This is not surprising given that *pccA* is widely distributed in all domains of life and is not unique to the 3HPB (64). *pccA* converts propionyl-CoA to acetyl-CoA, which can enter the Krebs cycle and generate succinate and three equivalents of NADH, a key process that utilizes small carbon molecules for energy generation for all organisms. Furthermore, several studies have shown that *Synechococcus* in alkaline hot springs release simple carbon compounds as a by-product of photosynthesis (6, 8, 12). Therefore, presence of several high and low abundance *pccA* reads, particularly in high temperature sites, is indicative of multiple organisms relying on the Krebs cycle to generate energy from simple carbon compounds at high temperatures.

Class Chlorobia contain type I reaction centers and are the only phototrophic group that fixes carbon via the rTCA cycle (4, 54). We recovered fewer reads associated with type I reaction centers (psc genes, Fig. S6A) compared to both type II reaction center and photosystem genes (Fig. 2). We recovered very few reads associated with either ATP citrate-lyase subunits, an irreversible and critical enzyme in the rTCA cycle. Together, these results suggest that phototrophic taxa with type I reaction centers are likely photoheterotrophs or photoautotrophs that use alternative carbon fixation pathways.

### (Putative) phototrophic Chloroflexi encode *nifH*.

**FIG 6:**
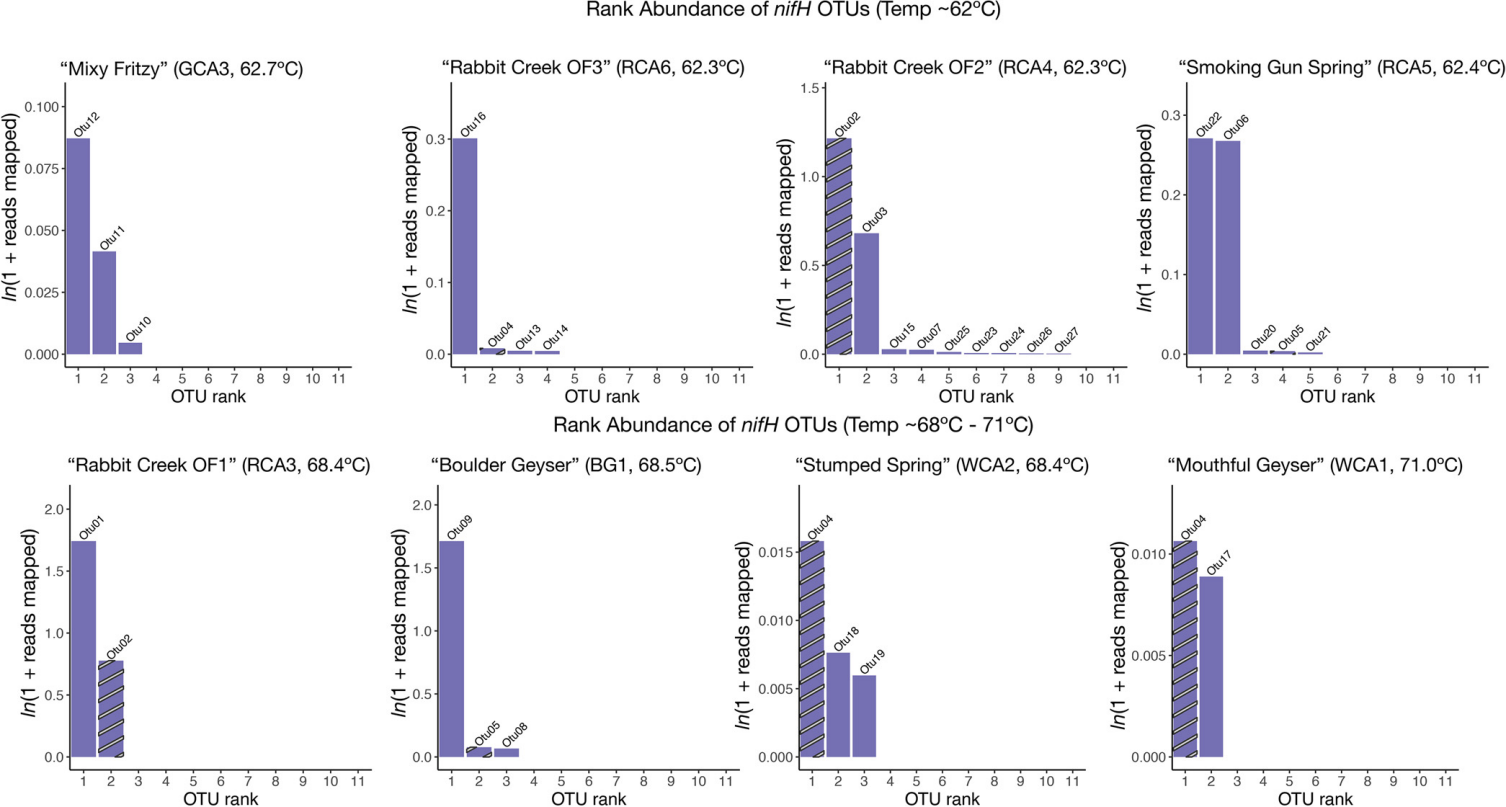
Richness and distribution of nifH gene variants. Rank abundance plots for each site are displayed in increasing temperature order. Plots display abundances as normalized ln(1 + reads mapped) for each nifH OTU, and OTUs are ranked in order from most to least abundant. Bars are labeled with the OTU number. Striped bars represent OTUs that are present in more than one site.

Alkaline hot springs in YNP are nitrogen limited, and several studies in Mushroom and Octopus Springs have shown that phototrophic bacteria are the primary diazotrophs in these environments (1, 4, 8, 29, 33). We examined the richness and diversity of nifH genes with respect to temperature (Fig. 6, Fig. S2C). Like our *psbA* and *pufLM* analysis above, we assigned OTUs (at 99% similarity) to the *nifH* sequences. We recovered 26 nifH OTUs, several of which were present in more than one site (Fig. 6). In general, we recovered more *nifH* OTUs in 62ºC sites (Fig. 6), but our most abundant OTU (assigned to *Synechococcus* sp. by BLASTP) was present in site RCA3 (68ºC). Sample GCA3 contained only unique OTUs, suggesting taxa with these *nifH* genes could be adapted to the distinct conditions in this site. Similarly, OTU05 was only present in the two high sulfide sites (RCA5 and BG1), and OTU04 was the most abundant in sites with the highest temperatures (WCA2 and WCA1). Our data suggest the potential for nitrogen fixation is not evenly distributed with temperature.

Loiacono et al. (2012) recovered *nifH* transcripts identified as *Synechococcus* and *Roseiflexus* in samples ranging from 53–73ºC, suggesting the potential for nitrogenase activity near the upper temperature limit of photosynthesis (33). To determine the taxa associated with our *nifH* sequences, we translated *nifH* sequences and built a phylogenetic tree and conducted a BLASTP search. Eleven of 26 *nifH* OTUs were classified as either cyanobacteria or Chloroflexi (Fig. S7A). Six *nifH* sequences were closely related to Synechococcus, a common constituent of alkaline hot springs of  >60ºC and a known diazotroph (Table in Fig. S7B) (30). Three of the 20 most abundant OTUs in our data set were closely related to *Roseiflexus* species (OTU02, 06, and 22), present in sites ranging from 62ºC to 68ºC in the Rabbit Creek area. *Roseiflexus* genomes only encode nifHBDK, and neither of the two isolate species (*R. castenholzii* or *Roseiflexus* sp. RS-1) can grow in the absence of a fixed nitrogen source (21, 65). Therefore, it is unlikely that *Roseiflexus* fixes nitrogen. However, *Roseiflexus nifH* genes are abundant in our data, and Roseiflexus nifH mRNA has been detected in similar hot springs (8, 15, 17, 30), suggesting NifH serves a functional purpose but that function remains unknown. In cyanobacteria, NifH expression is stimulated by iron (66). Our samples ranged in Fe^2+^ concentration from below detection limits to 2.3 μM but given that *Roseiflexus* genomes don’t encode a full nitrogenase, future studies are required to determine the function of NifH in this genus and the conditions that result in transcription. *Roseiflexus nifH* could also be important to determining the evolutionary history of nitrogenase as *Roseiflexus nif* genes are deeply branching (67).

The second most abundant *nifH* OTU in our data set (OTU09) formed a separate clade near, but not within, the cyanobacteria clade (Fig. S7A). BLASTP assigned OTU09 (and four additional, low abundance OTUs; Table in Fig. S6B) as Hydrogenobacter thermophilus, in phylum Aquificae, a deep-branching chemolithoautotrophic group with diazotrophic representatives found in high temperature (>70ºC) hot springs (68). Previous analysis of *nifH* genes across all domains of life suggested Aquificae are the oldest extant diazotrophic bacteria (26). Thus, our data contain several *nifH*-containing lineages that are of great importance for solving the evolutionary history of nitrogen fixation.

### Conclusion.

Phototrophic bacteria are widely distributed and abundant in alkaline hot springs at  >60ºC. By quantifying the distribution of genes involved in carbon fixation, nitrogen fixation, and phototrophy in eight alkaline hot spring metagenomes, we add to the large body of work on the metabolic potential of both cyanobacteria and anoxygenic phototrophs *in situ*. Additionally, we offer a glimpse into the diversity and physiology of the underrepresented Chloroflexi phylum. While the abundance of photosynthetic genes did not vary with temperature, we observed higher richness in both cyanobacterial *psbA* genes and *pufLM* genes affiliated with Chloroflexi in 62ºC sites. Furthermore, we observed more cosmopolitan *psbA* OTUs in 62ºC sites and unique OTUs in sites of > 68ºC. This suggests that cyanobacteria at higher temperatures contain forms of *psbA* genes that could allow them to persist at higher temperatures. Conversely, we observed several cosmopolitan *pufLM* OTUs in both high and low temperature sites, specifically OTUs shared across the Rabbit Creek area, which suggest Chloroflexi are adapted to local geothermal conditions rather than specific temperatures.

Abundance of photosynthesis genes associated with both cyanobacteria and phototrophic Chloroflexi did not significantly differ with temperature. Carbon fixation gene abundances were significantly different in site RCA5 compared to all others. However, in general, we did not observe trends in abundance with temperature. Rather, ratios of *rbcL* genes suggest temperature selects for specific types of RuBisCO: cyanobacterial-*rbcL* in sites >63ºC and noncyanobacterial-*rbcL* in 62ºC sites. Furthermore, the majority of the rbcL OTUs were unique to certain sites, suggesting geographic isolation or adaptation to local conditions. Genes associated with autotrophic, anoxygenic phototrophs did not have distinct distributions with temperature, but we recovered abundant reads associated with the 3-hydroxypropionate bicycle (Chloroflexi, chemoautotrophs) and very few reads associated with the complete reverse TCA cycle (Chlorobia). Together, abundance and diversity of carbon fixation genes suggest that organisms fixing CO2 via the rTCA cycle are rare near the upper temperature limit of photosynthesis where photoautotrophic cyanobacteria and Chloroflexi are abundant.

Finally, we surveyed the distribution and abundance of genes associated with nitrogen fixation (*nifH*). *NifH* genes were abundant across sites, regardless of site temperature, and both *Roseiflexus* and *Synechococcus*-like *nifH* sequences were among the most abundant in our data. *Synechococcus* are known to fix nitrogen in hot springs, but *Roseiflexus* do not have the full suite of genes required to fix nitrogen; yet, *nifH*-containing *Roseiflexus* are abundant in alkaline hot springs, and Chloroflexi are deep-branching taxa. Thus, *nifH* sequences recovered here could be critical to solving the evolutionary puzzle of nitrogen fixation in bacteria.

## MATERIALS AND METHODS

### Data collection, sample processing, and metadata statistics.

Biomass from eight sites in YNP (Table S1A) were collected and processed as previously described (11). Briefly, samples were collected in 2017 using sterilized forceps or pliers and stored on dry ice in transit. DNA (250 mg) was extracted using the Qiagen Powersoil kit following the manufacturer’s protocol. Sulfide, Fe^2+^, and dissolved silica were measured onsite using a DR1900 portable spectrophotometer (Hach Company, Loveland, CO). Water samples were filtered through 0.2-μm polyethersulfone syringe filters (VWR International, Radnor, PA, USA) and analyzed for dissolved inorganic carbon (DIC) concentration, δ^13^C and δ^15^N as described previously (25). Field blanks composed of filtered 18.2 MΩ/cm deionized water, transported to the field in 1-L Nalgene bottles, were processed on site using the equipment and techniques previously described (11). To determine site dissimilarity, we generated a principal-component analysis using sample water geochemistry, geographic location, and biofilm isotopic data (Table S1A) (11). We converted all raw data to Z-scores (z = x – mean(x)/sd(x)), and principal components of transformed data were generated using the rda function in vegan (69) and plotted using ggplot2.

### Metagenome sequencing, assembly, and analysis of functional genes.

Total DNA for eight samples was submitted to the University of Minnesota Genomics Center (St. Paul, MN, UMGC) for metagenomic sequencing with an Illumina HiSeq 2500. The UMGC prepared dual indexed Nextera XT DNA libraries following the manufacturer’s instructions for each sample. The samples were sequenced on two lanes, generating >220M 1 × 125 bp reads. The mean quality scores were >Q30 for all libraries. Reads were trimmed using Sickle (v. 1.33) with a PHRED SCOREof  >20 and a minimum length threshold of 50 (70), assembled using SPades (v. 3.11.0) (71) using the meta option and default parameters, and assessed for quality using the BBTools script stats.sh (72).

Metagenome assemblies for eight sites (Table S1) were submitted to the Joint Genome Institute for structural and functional annotation via the DOE-JGI Microbial Genome Annotation Pipeline (https://img.jgi.doe.gov/). Briefly, open reading frames (ORFs) were predicted using Prodigal (73) and the resulting amino acid sequences were assigned functional annotations. Select genes (see supplemental material) involved in three carbon fixation pathways (the Calvin Cycle, 3-Hydroxypriopionate Bicycle, and the reverse Tricarboxylic Acid cycle), nitrogen fixation, and photosynthesis were queried in the annotated assemblies. Genes of interest were retrieved using known functional KEGG Orthologies. Metagenome reads were mapped to each JGI ORF using Bowtie2 (74). Reads that mapped to >90% of the query length sequence at 100% sequence identity were considered mapped. The average number of reads in the eight metagenomes was 830,473, with a standard deviation of 267,811 reads (Table S1B). In our metagenome assemblies, the maximum number of reads was from site WCA1 (1,187,870 reads), while the lowest number of reads was from site RCA5 (375,420 reads) (Table S1B). Site RCA4 contained the highest number genes, 332,336, while site RCA5 had the lowest number of genes, 150,190 (Table S1B).

To determine abundance of select genes involved in photosynthesis, carbon fixation, and nitrogen fixation, number of reads mapped to genes of interest was calculated using the pileup.sh script in BBTools (72). In order to directly compare genes of interest, genes were normalized by gene length and metagenome size using the following equation:

reads mapped to genegenelength×(1Σreads mapped to each genelength of each gene)×106

If multiple ORFs were assigned to a functional annotation, the normalized read abundance for that functional annotation was averaged. All analysis of functional genes, plotting, and statistical analysis was conducted in R (v. 3.6.1) (75) using the following packages: tidyverse (76), ggplot2 (64), vegan (63), and lawstat (77). To determine significant differences of normalized gene abundances across sites, a Kruskal-Wallis H test followed by Dunn’s Multiple Comparison *post hoc* test for significant differences between sites was conducted. *P* values were Bonferroni adjusted and are displayed in the supplemental information.

### Gene operational taxonomic unit (OTU) assignment and gene tree construction.

To determine the distribution of gene variants in our metagenomes, DNA reference sequences for *psbA* (see supplemental material), *rbcL* (see supplemental material), *nifH* (78), and concatenated *pufLM* (44) were downloaded, aligned using MUSCLE v. 3.8.31(default parameters), (79) and aligned with sample DNA sequences using align.seqs() in mothur (v.1.37.6) (80). Operational taxonomic units (OTUs, defined at 99% sequence identity, 28) were assigned using pre.cluster(), dist.seqs(), and cluster() in mothur. To generate protein sequences for phylogenetic tree construction, OTUs were translated using the transeq function in emboss (v. 6.5.7.0) (81), sequences of less than 200 amino acids were removed, sequences were aligned with MUSCLE v. 3.8.31(default parameters) (79), and alignments were trimmed using Gblocks (default parameters with the exception of -b5-h) (82). Phylogenetic analysis with bootstrap support (*n* = 1000) of trimmed, aligned protein sequences was conducted using RAxML (v. 8.2.11) using the PROTGAMMAJTTF substitution model, following the RAxML SOP (83). The subsequent newick file was edited using FigTree (v. 1.4.4) (84) to generate trees. Because of low bootstrap support due to closely related species in all three of our phylogenetic trees, we conducted a BLASTP search (nonredundant protein sequences) (47) to determine closest relatives of our OTUs. For the *nifH* OTUs, specifically, we aligned the metal binding subunit retrieved from Uniprot (85) to show functionality using the program MUSCLE (73).

### Data availability.

Access to the metagenomes is provided by the DOE Joint Genome Institute (JGI) at the Integrated Microbial Genome (IMG-M) site: https://img.jgi.doe.gov/cgi-bin/m/main.cgi. JGI Genome IDs are provided in Table S1. Quality-controlled, unassembled, metagenomic data are available in the NCBI Sequence Read Archive under the project ID PRJNA513338.

## References

[1] Thiel V, Tank M, Bryant DA. 2018. Diversity of chlorophototrophic bacteria revealed in the Omics Era. Annu Rev Plant Biol 69:21–49. doi:10.1146/annurev-arplant-042817-040500. 29505738

[2] Inskeep WP, Jay ZJ, Tringe SG, Herrgard MJ, Rusch DB. 2013. The YNP metagenome project: environmental parameters responsible for microbial distribution in the Yellowstone geothermal ecosystem. Front Microbiol 4:67. 23653623 10.3389/fmicb.2013.00067 PMC3644721

[3] Ward DM, Cohan FM. 2005. Microbial diversity in hot spring cyanobacterial mats: pattern and prediction, p 185–202. *In* Inskeep WP, McDermott T (ed), Geothermal biology and geochemistry in Yellowstone National Park. Thermal Biology Institute, Bozeman, Montana.

[4] Tank M, Thiel V, Ward DM, Bryant DA. 2017. A panoply of phototrophs: an overview of the thermophilic chlorophototrophs of the microbial mats of alkaline siliceous hot springs in Yellowstone National Park, WY, USA, p 87–137. *In* Hallenbeck P (ed), Modern topics in the phototrophic prokaryotes: environmental and applied aspects. Springer, New York, NY.

[5] Ward DM, Ferris MJ, Nold SC, Bateson MM. 1998. A natural view of microbial biodiversity within hot spring cyanobacterial mat communities. Microbiol Mol Biol Rev 62:1353–1370. doi:10.1128/MMBR.62.4.1353-1370.1998. 9841675 PMC98949

[6] Becraft ED, Cohan FM, Kühl M, Jensen SI, Ward DM. 2011. Fine-scale distribution patterns of Synechococcus ecological diversity in microbial mats of Mushroom Spring, Yellowstone National Park. Appl Environ Microbiol 77:7689–7697. doi:10.1128/AEM.05927-11. 21890675 PMC3209189

[7] Klatt CG, Wood JM, Rusch DB, Bateson MM, Hamamura N, Heidelberg JF, Grossman AR, Bhaya D, Cohan FM, Kühl M, Bryant DA, Ward DM. 2011. Community ecology of hot spring cyanobacterial mats: predominant populations and their functional potential. ISME J 5:1262–1278. doi:10.1038/ismej.2011.73. 21697961 PMC3146275

[8] Klatt CG, Liu Z, Ludwig M, Kühl M, Jensen SI, Bryant DA, Ward DM. 2013. Temporal metatranscriptomic patterning in phototrophic Chloroflexi inhabiting a microbial mat in a geothermal spring. ISME J 7:1775–1789. doi:10.1038/ismej.2013.52. 23575369 PMC3749495

[9] Miller SR, Strong AL, Jones KL, Ungerer MC. 2009. Bar-coded pyrosequencing reveals shared bacterial community properties along the temperature gradients of two alkaline hot springs in Yellowstone National Park. Appl Environ Microbiol 75:4565–4572. doi:10.1128/AEM.02792-08. 19429553 PMC2704827

[10] Bennett AC, Murugapiran SK, Hamilton TL. 2020. Temperature impacts community structure and function of phototrophic Chloroflexi and Cyanobacteria in two alkaline hot springs in Yellowstone National Park. Environ Microbiol Rep 12:503–513. doi:10.1111/1758-2229.12863. 32613733 PMC7540483

[11] Hamilton TL, Bennett AC, Murugapiran SK, Havig JR. 2019. Anoxygenic phototrophs span geochemical gradients and diverse morphologies in terrestrial geothermal springs. mSystems 4:e00498-19. Available from: http://msystems.asm.org/content/4/6/e00498-19 doi:10.1128/mSystems.00498-19. 31690593 PMC6832021

[12] Nübel U, Bateson MM, Vandieken V, Wieland A, Kühl M, Ward DM. 2002. Microscopic examination of distribution and phenotypic properties of phylogenetically diverse *Chloroflexaceae*-related bacteria in hot spring microbial mats. Appl Environ Microbiol 68:4593–4603. doi:10.1128/AEM.68.9.4593-4603.2002. 12200318 PMC124081

[13] Schuler CG, Havig JR, Hamilton TL. 2017. Hot spring microbial community composition, morphology, and carbon fixation: implications for interpreting the ancient rock record. Front Earth Sci 5:1–17.

[14] Havig JR, Raymond J, Meyer-Dombard DR, Zolotova N, Shock EL. 2011. Merging isotopes and community genomics in a siliceous sinter-depositing hot spring. J Geophys Res Biogeosciences 116:1–15.

[15] Alcorta J, et al. 2020. Taxonomic novelty and distinctive genomic features of hot spring cyanobacteria. Front Genet 11:568223. 33250920 10.3389/fgene.2020.568223 PMC7674949

[16] de Beer D, Weber M, Chennu A, Hamilton T, Lott C, Macalady J, M Klatt J. 2017. Oxygenic and anoxygenic photosynthesis in a microbial mat from an anoxic and sulfidic spring. Environ Microbiol 19:1251–1265. doi:10.1111/1462-2920.13654. 28035767

[17] Thiel V, et al. 2016. The dark side of the Mushroom spring microbial mat: life in the shadow of chlorophototrophs. I. Microbial diversity based on 16S rRNA gene amplicons and metagenomic sequencing. Front Microbiol 7:919. 27379049 10.3389/fmicb.2016.00919 PMC4911352

[18] Martinez JN, Kawai S, Saini MK, Tank M, Hanada S, Thiel V. 2020. Draft genome sequence of a filamentous anoxygenic phototrophic bacterium, “*Candidatus* Roseilinea sp. Strain NK_OTU-006,” recovered from metagenomic data of a hot spring microbial mat. Microbiol Resour Announc 9:18–20. doi:10.1128/MRA.01104-20. PMC7729410 33303662

[19] Ward LM, Fatima L, Kakegawa T, McGlynn SE. 2020. Complex history of aerobic respiration and phototrophy in the Chloroflexota class Anaerolineae revealed by high-quality draft genome of *Ca*. Roseilinea mizusawaensis AA3_104. bioRxiv. doi:10.1101/2020.11.30.404129. PMC8446752 34470945

[20] Liu Z, Klatt CG, Wood JM, Rusch DB, Ludwig M, Wittekindt N, Tomsho LP, Schuster SC, Ward DM, Bryant DA. 2011. Metatranscriptomic analyses of chlorophototrophs of a hot-spring microbial mat. ISME J 5:1279–1290. doi:10.1038/ismej.2011.37. 21697962 PMC3146272

[21] Hanada S, Takaichi S, Matsuura K, Nakamura K. 2002. *Roseiflexus castenholzii* gen. nov., sp. nov., a thermophilic, filamentous, photosynthetic bacterium that lacks chlorosomes. Int J Syst Evol Microbiol 52:187–193. doi:10.1099/00207713-52-1-187. 11837302

[22] Hanada S, Hiraishi A, Shimada K, Matsuura K. 1995. *Chloroflexus aggregans* sp. nov., a filamentous phototrophic bacterium which forms dense cell aggregates by active gliding movement. Int J Syst Bacteriol 45:676–681. doi:10.1099/00207713-45-4-676. 7547286

[23] Madigan MT, Brock TD. 1975. Photosynthetic sulfide oxidation by *Chloroflexus aurantiacus*, a filamentous, photosynthetic, gliding bacterium. J Bacteriol 122:782–784. doi:10.1128/jb.122.2.782-784.1975. 1092670 PMC246117

[24] Kawai S, Martinez JN, Lichtenberg M, Trampe E, Kühl M, Tank M, Haruta S, Nishihara A, Hanada S, Thiel V. 2021. *In-situ* metatranscriptomic analyses reveal the metabolic flexibility of the thermophilic anoxygenic photosynthetic bacterium *Chloroflexus aggregans* in a hot spring cyanobacteria-dominated microbial mat. Microorganisms 9:652. doi:10.3390/microorganisms9030652. 33801086 PMC8004040

[25] Swingley WD, Meyer-Dombard DR, Shock EL, Alsop EB, Falenski HD, Havig JR, Raymond J. 2012. Coordinating environmental genomics and geochemistry reveals metabolic transitions in a hot spring ecosystem. PLoS One 7:e38108. doi:10.1371/journal.pone.0038108. 22675512 PMC3367023

[26] Boyd ES, Peters JW. 2013. New insights into the evolutionary history of biological nitrogen fixation. Front Microbiol 4:201–212. 23935594 10.3389/fmicb.2013.00201 PMC3733012

[27] McGlynn SE, et al. 2012. Classifying the metal dependence of uncharacterized nitrogenases. Front Microbiol 3:419. 23440025 10.3389/fmicb.2012.00419 PMC3578447

[28] Thakur S, Bothra AK, Sen A. 2013. Functional divergence outlines the evolution of novel protein function in NifH/BchL protein family. J Biosci 38:733–740. doi:10.1007/s12038-013-9360-5. 24287653

[29] Hamilton TL, Lange RK, Boyd ES, Peters JW. 2011. Biological nitrogen fixation in acidic high-temperature geothermal springs in Yellowstone National Park, Wyoming. Environ Microbiol 13:2204–2215. doi:10.1111/j.1462-2920.2011.02475.x. 21450003

[30] Hamilton TL, Boyd ES, Peters JW. 2011. Environmental constraints underpin the distribution and phylogenetic diversity of nifH in the Yellowstone geothermal complex. Microb Ecol 61:860–870. doi:10.1007/s00248-011-9824-9. 21365232

[31] Alcamán-Arias ME, et al. 2018. Diurnal changes in active carbon and nitrogen pathways along the temperature gradient in Porcelana hot spring microbial mat. Front Microbiol 9:2353. doi:10.3389/fmicb.2018.02353. 30333812 PMC6176055

[32] Steunou A-S, Jensen SI, Brecht E, Becraft ED, Bateson MM, Kilian O, Bhaya D, Ward DM, Peters JW, Grossman AR, Kühl M. 2008. Regulation of *nif* gene expression and the energetics of N_2_ fixation over the diel cycle in a hot spring microbial mat. ISME J 2:364–378. doi:10.1038/ismej.2007.117. 18323780

[33] Loiacono ST, Meyer-Dombard DR, Havig JR, Poret-Peterson AT, Hartnett HE, Shock EL. 2012. Evidence for high-temperature *in situ nifH* transcription in an alkaline hot spring of Lower Geyser Basin, Yellowstone National Park. Environ Microbiol 14:1272–1283. doi:10.1111/j.1462-2920.2012.02710.x. 22404902

[34] Galambos D, Anderson RE, Reveillaud J, Huber JA. 2019. Genome-resolved metagenomics and metatranscriptomics reveal niche differentiation in functionally redundant microbial communities at deep-sea hydrothermal vents. Environ Microbiol 21:4395–4410. doi:10.1111/1462-2920.14806. 31573126 PMC6899741

[35] Papke RT, Ramsing NB, Bateson MM, Ward DM. 2003. Geographical isolation in hot spring cyanobacteria. Environ Microbiol 5:650–659. doi:10.1046/j.1462-2920.2003.00460.x. 12871232

[36] Becraft ED, et al. 2020. Biogeography of American Northwest hot spring A/B′-lineage Synechococcus populations. Front Microbiol 11:77. doi:10.3389/fmicb.2020.00077. 32153516 PMC7050468

[37] Mulo P, Sicora C, Aro E-M. 2009. Cyanobacterial psbA gene family: optimization of oxygenic photosynthesis. Cell Mol Life Sci 66:3697–3710. doi:10.1007/s00018-009-0103-6. 19644734 PMC2776144

[38] Mulo P, Sakurai I, Aro E-M. 2012. Strategies for psbA gene expression in cyanobacteria, green algae and higher plants: from transcription to PSII repair. Biochim Biophys Acta 1817:247–257. doi:10.1016/j.bbabio.2011.04.011. 21565160

[39] Garczarek L, Dufresne A, Blot N, Cockshutt AM, Peyrat A, Campbell DA, Joubin L, Six C. 2008. Function and evolution of the psbA gene family in marine Synechococcus: Synechococcus sp. WH7803 as a case study. ISME J 2:937–953. doi:10.1038/ismej.2008.46. 18509382

[40] Gan F, Bryant DA. 2015. Adaptive and acclimative responses of cyanobacteria to far-red light. Environ Microbiol 17:3450–3465. doi:10.1111/1462-2920.12992. 26234306

[41] Altschul SF, Gish W, Miller W, Myers EW, Lipman DJ. 1990. Basic local alignment search tool. J Mol Biol 215:403–410. doi:10.1016/S0022-2836(05)80360-2. 2231712

[42] Becraft ED, et al. 2015. The molecular dimension of microbial species: 2. Synechococcus strains representative of putative ecotypes inhabiting different depths in the Mushroom Spring microbial mat exhibit different adaptive and acclimative responses to light. Front Microbiol 6:590. 26175719 10.3389/fmicb.2015.00626 PMC4484337

[43] Kees ED, Murugapiran SK, Bennett AC, Hamilton TL. 2022. Distribution and genomic variation of thermophilic cyanobacteria in diverse microbial mats at the upper temperature limits of photosynthesis. bioRxiv. doi:10.1101/2022.03.25.485844. PMC9600594 35980085

[44] Pedersen D, Miller SR. 2017. Photosynthetic temperature adaptation during niche diversification of the thermophilic cyanobacterium Synechococcus A/B clade. ISME J 11:1053–1057. 4. doi:10.1038/ismej.2016.173. 27983722 PMC5364356

[45] Imhoff JF, et al. 2018. Photosynthesis is widely distributed among Proteobacteria as demonstrated by the phylogeny of PufLM reaction center proteins. Front Microbiol 8:2679. doi:10.3389/fmicb.2017.02679. 29472894 PMC5810265

[46] Tsukatani Y, Matsuura K, Masuda S, Shimada K, Hiraishi A, Nagashima KVP. 2004. Phylogenetic distribution of unusual triheme to tetraheme cytochrome subunit in the reaction center complex of purple photosynthetic bacteria. Photosynth Res 79:83–91. doi:10.1023/B:PRES.0000011922.56394.92. 16228402

[47] Kondrashov FA. 2012. Gene duplication as a mechanism of genomic adaptation to a changing environment. Proc Biol Sci 279:5048–5057. 22977152 10.1098/rspb.2012.1108 PMC3497230

[48] Thiel V, et al. 2017. The dark side of the Mushroom Spring microbial mat: Life in the shadow of chlorophototrophs. II. Metabolic functions of abundant community members predicted from metagenomic analyses. Front Microbiol 8:943. doi:10.3389/fmicb.2017.00943. 28634470 PMC5459899

[49] Giovannoni SJ, Revsbech NP, Ward DM, Castenholz RW. 1987. Obligately phototrophic Chloroflexus: primary production in anaerobic hot spring microbial mats. Arch Microbiol 147:80–87. doi:10.1007/BF00492909.

[50] Ruff-Roberts AL, Kuenen JG, Ward DM. 1994. Distribution of cultivated and uncultivated cyanobacteria and Chloroflexus-like bacteria in hot spring microbial mats. Appl Environ Microbiol 60:697–704. doi:10.1128/aem.60.2.697-704.1994. 11536630 PMC201368

[51] Pierson BK, Castenholz RW. 1974. A phototrophic gliding filamentous bacterium of hot springs, *Chloroflexus aurantiacus*, gen. and sp. nov. Arch Microbiol 100:5–24. doi:10.1007/BF00446302. 4374148

[52] Brock TD. 1967. Micro-organisms adapted to high temperatures. Nature 214:882–885. doi:10.1038/214882a0. 6054968

[53] Castenholz RW. 1978. The biogeography of hot spring algae through enrichment cultures. SIL Commun 1953–1996 21:296–315. doi:10.1080/05384680.1978.11903973.

[54] Fuchs G. 2011. Alternative pathways of carbon dioxide fixation: insights into the early evolution of life? Annu Rev Microbiol 65:631–658. doi:10.1146/annurev-micro-090110-102801. 21740227

[55] Ward LM, Idei A, Nakagawa M, Ueno Y, Fischer WW, McGlynn SE. 2019. Geochemical and metagenomic characterization of Jinata Onsen, a proterozoic-analog hot spring, reveals novel microbial diversity including iron-tolerant phototrophs and thermophilic lithotrophs. Microbes Environ 34:278–292. doi:10.1264/jsme2.ME19017. 31413226 PMC6759342

[56] Tsuji JM, et al. 2020. Type I photosynthetic reaction center in an anoxygenic phototrophic member of the *Chloroflexota*. bioRxiv doi:10.1101/2020.07.07.190934.

[57] Ward LM, et al. 2019. Evolutionary implications of anoxygenic phototrophy in the bacterial phylum *Candidatus* Eremiobacterota (WPS-2). Front Microbiol 10:1658. doi:10.3389/fmicb.2019.01658. 31396180 PMC6664022

[58] Miller SR, McGuirl MA, Carvey D. 2013. The evolution of RuBisCO stability at the thermal limit of photoautotrophy. Mol Biol Evol 30:752–760. doi:10.1093/molbev/mss327. 23292343

[59] Tabita FR, Hanson TE, Li H, Satagopan S, Singh J, Chan S. 2007. Function, structure, and evolution of the RubisCO-like proteins and their RubisCO homologs. Microbiol Mol Biol Rev 71:576–599. doi:10.1128/MMBR.00015-07. 18063718 PMC2168653

[60] Ellis RJ. 1979. The most abundant protein in the world. Trends Biochem Sci 4:241–244. doi:10.1016/0968-0004(79)90212-3.

[61] Tabita FR, Satagopan S, Hanson TE, Kreel NE, Scott SS. 2007. Distinct form I, II, III, and IV Rubisco proteins from the three kingdoms of life provide clues about Rubisco evolution and structure/function relationships. J Exp Bot 59:1515–1524. doi:10.1093/jxb/erm361. 18281717

[62] Frolov EN, Kublanov IV, Toshchakov SV, Lunev EA, Pimenov NV, Bonch-Osmolovskaya EA, Lebedinsky AV, Chernyh NA. 2019. Form III RubisCO-mediated transaldolase variant of the Calvin cycle in a chemolithoautotrophic bacterium. Proc Natl Acad Sci USA 116:18638–18646. doi:10.1073/pnas.1904225116. 31451656 PMC6744853

[63] Zarzycki J, Brecht V, Müller M, Fuchs G. 2009. Identifying the missing steps of the autotrophic 3-hydroxypropionate CO2 fixation cycle in *Chloroflexus aurantiacus*. Proc Natl Acad Sci USA 106:21317–21322. doi:10.1073/pnas.0908356106. 19955419 PMC2795484

[64] Hou J, Xiang H, Han J. 2015. Propionyl coenzyme A (Propionyl-CoA) carboxylase in *Haloferax mediterranei*: indispensability for propionyl-CoA assimilation and impacts on global metabolism. Appl Environ Microbiol 81:794–804. doi:10.1128/AEM.03167-14. 25398867 PMC4277572

[65] van der Meer MTJ, Klatt CG, Wood J, Bryant DA, Bateson MM, Lammerts L, Schouten S, Damsté JSS, Madigan MT, Ward DM. 2010. Cultivation and genomic, nutritional, and lipid biomarker characterization of Roseiflexus strains closely related to predominant in situ populations inhabiting Yellowstone hot spring microbial mats. J Bacteriol 192:3033–3042. doi:10.1128/JB.01610-09. 20363941 PMC2901690

[66] Turk-Kubo KA, Achilles KM, Serros TRC, Ochiai M, Montoya JP, Zehr JP. 2012. Nitrogenase (nifH) gene expression in diazotrophic cyanobacteria in the Tropical North Atlantic in response to nutrient amendments. Front Microbiol 3:386. 23130017 10.3389/fmicb.2012.00386 PMC3487379

[67] Boyd ES, Anbar AD, Miller S, Hamilton TL, Lavin M, Peters JW. 2011. A late methanogen origin for molybdenum-dependent nitrogenase. Geobiology 9:221–232. doi:10.1111/j.1472-4669.2011.00278.x. 21504537

[68] Nishihara A, Matsuura K, Tank M, McGlynn SE, Thiel V, Haruta S. 2018. Nitrogenase activity in thermophilic chemolithoautotrophic bacteria in the phylum Aquificae isolated under nitrogen-fixing conditions from Nakabusa hot springs. Microbes Environ 33:394–401. doi:10.1264/jsme2.ME18041. 30473565 PMC6307999

[69] Oksanen J. 2009. The vegan package. Community Ecology Package 10:719.

[70] Joshi NA, Fass JN. 2011. Sickle: a sliding-window, adaptive, quality-based trimming tool for FastQ files (Version 1.33). https://github.com/najoshi/sickle.

[71] Bankevich A, Nurk S, Antipov D, Gurevich AA, Dvorkin M, Kulikov AS, Lesin VM, Nikolenko SI, Pham S, Prjibelski AD, Pyshkin AV, Sirotkin AV, Vyahhi N, Tesler G, Alekseyev MA, Pevzner PA. 2012. SPAdes: a new genome assembly algorithm and its applications to single-cell sequencing. J Comput Biol 19:455–477. doi:10.1089/cmb.2012.0021. 22506599 PMC3342519

[72] Bushnell B. BBMap. sourceforge.net/projects/bbmap/.

[73] Hyatt D, Chen G-L, Locascio PF, Land ML, Larimer FW, Hauser LJ. 2010. Prodigal: prokaryotic gene recognition and translation initiation site identification. BMC Bioinformatics 11:119. doi:10.1186/1471-2105-11-119. 20211023 PMC2848648

[74] Langmead B, Salzberg SL. 2012. Fast gapped-read alignment with Bowtie 2. Nat Methods 9:357–359. doi:10.1038/nmeth.1923. 22388286 PMC3322381

[75] Core Team R. 2014. R: A language and environment for statistical computing. R Foundation for Statistical Computing, Vienna, Austria. http://www.R-project.org/.

[76] Wickham H, Averick M, Bryan J, Chang W, McGowan L, François R, Grolemund G, Hayes A, Henry L, Hester J, Kuhn M, Pedersen T, Miller E, Bache S, Müller K, Ooms J, Robinson D, Seidel D, Spinu V, Takahashi K, Vaughan D, Wilke C, Woo K, Yutani H. 2019. Welcome to the tidyverse. Joss 4:1686. doi:10.21105/joss.01686.

[77] Gastwirth JL, et al. 2020. lawstat: tools for biostatistics, public policy, and law. https://rdrr.io/cran/lawstat/.

[78] Gaby JC, Buckley DH. 2014. A comprehensive aligned nifH gene database: a multipurpose tool for studies of nitrogen-fixing bacteria. Database 2014:bau001. 10.1093/database/bau001. 24501396 PMC3915025

[79] Edgar RC. 2004. MUSCLE: multiple sequence alignment with high accuracy and high throughput. Nucleic Acids Res 32:1792–1797. doi:10.1093/nar/gkh340. 15034147 PMC390337

[80] Oakley BB, et al. 2009. Introducing mothur: open-source, platform-independent, community-supported software for describing and comparing microbial communities. Appl Environ Microbiol 15:7537–7541. 10.1128/AEM.01541-09 PMC2786419 19801464

[81] Rice P, Longden I, Bleasby A. 2000. EMBOSS: the European molecular biology open software suite. Trends Genet 16:276–277. doi:10.1016/S0168-9525(00)02024-2. 10827456

[82] Castresana J. 2000. Selection of conserved blocks from multiple alignments for their use in phylogenetic analysis. Mol Biol Evol 17:540–552. doi:10.1093/oxfordjournals.molbev.a026334. 10742046

[83] Stamatakis A. 2014. RAxML version 8: a tool for phylogenetic analysis and post-analysis of large phylogenies. Bioinformatics 30:1312–1313. 24451623 10.1093/bioinformatics/btu033 PMC3998144

[84] Drummond AJ. 2017. FigTree. https://beast.community/figtree.

[85] Bateman A. 2019. UniProt: a worldwide hub of protein knowledge. Nucleic Acids Res 47:D506–D515. 30395287 10.1093/nar/gky1049 PMC6323992

