# Supplementary material for: Temperature and Geographic Location Impact the Distribution and Diversity of Photoautotrophic Gene Variants in Alkaline Yellowstone Hot Springs
Source: Microbiol Spectr. 2022 May 16;10(3):e01465-21. doi: 10.1128/spectrum.01465-21 (PMC9241655; doi:10.1128/spectrum.01465-21)
Supplement: SUPPLEMENTAL FILE 1 — Supplemental material. Download spectrum.01465-21-s001.pdf, PDF file, 1.8 MB [file spectrum.01465-21-s001.pdf]

SUPPLEMENTAL MAERIAL FOR PUBLICATION

Manuscript title: Temperature and geographic location impact the distribution, diversity, and abundance of photoautotrophic gene variants in alkaline Yellowstone Hot Springs

Author list: Annastacia C. Bennett,[a] Senthil K. Murugapiran,[a,†] Eric D. Kees,[a] Hailey M. Sauer, [a] Trinity L. Hamilton[a,b]#

Supplemental figure legends

Table S1. Sample site and metagenome meta data.

(A) Sample IDs and physicochemical parameters of sample sites. pH, conductivity, temperature, and aqueous geochemistry of spring water at the site of sample collection. bdl, below detection limit. Detection limits: Fe^{2+}, 20 μg/L. (B) Metagenome assembly statistics. Sample IDs and metagenome meta-data. NCBI Accession numbers, JGI genome IDs, number of genes identified (ORFs), number of reads, N50, and number of contigs for each metagenome.

Figure S1. Maximum likelihood-inferred PsbA tree and best BLASTP match.

A) Maximum likelihood-inferred phylogenetic reconstruction of translated *psbA* OTUs recovered from YNP sites (bold) aligned to all reference sequences in Supplemental File 1. NCBI taxonomy IDs are shown for each uncollapsed reference taxon. The tree is rooted with a marine Cyanobacterium, *Prochlorococcus marinus* PsbA. The scale bar indicates 4 substitutions for every ten positions. Bootstrap values (n=1000 bootstrap replicates) are displayed at branches. Bootstrap values less than 50 are not shown. **Collapsed clades:** *Synecococcus* Clade 1 (*Synechococcus* sp. MIT9509, *Synechococcus* sp. KORDI-52, *Synechococcus* sp. WH 8109, *Synechococcus* sp. 1G10); *Synecococcus* Clade 2 (*Synechococcus* sp. WH 8020, *Synechococcus* sp. CC9902, *Synechococcus* sp. WH8103, *Synechococcus* sp. CC9605, *Synechococcus* sp. KORDI-49, *Synechococcus* sp. MIT9504, *Synechococcus* sp. WH 7803, *Synechococcus* sp. KORDI-100, *Synechococcus* sp. RSCCF101); *Leptolynbya* sp. (Leptolyngbya sp. KIOST-1, Leptolyngbya sp. BC1307); *Synechocystis* sp. (Synechocystis sp. PCC 6803, Synechocystis sp. PCC 6803, PCC-P); *Synecococcus* Clade 3 (*Synechococcus* sp. PCC 7002, *Synechococcus* sp. WH 7803); *Leptolynbya* Clade 2 (*Leptolyngbya* boryana dg5, *Leptolyngbya* sp. NIES-3755); *Synechococcus* & *Psuedoanabaena* clade (*Synechococcus* sp. PCC 7502, *Pseudanabaena* sp. PCC 7367). (B) Abundance and best BLASTP hit for all *psbA* OTUs.

Figure S2. Alpha diversity of *psbA, pufLM, nifH,* and *rbcL* gene variants.

Shannon diversity of (A) *psbA,* (B) *pufLM,* (C) *nifH,* and (D) *rbcL* gene variants for each site, divided into high and low temperature groups, and arranged by increasing temperature.

Figure S3. Maximum likelihood-inferred PufLM tree, OTU best BLASTP match, and PufLM BLASTP counts for *Roseiflexus* and *Chloroflexus* genera.

A) Maximum likelihood-inferred phylogenetic reconstruction of unique concatenated, translated *pufLM* OTUs recovered from YNP sites (bold) aligned to all reference sequences used the reference 2. NCBI taxonomy IDs are shown for each uncollapsed reference taxon. The tree is rooted with *methylobacterium sp* PufLM. The scale bar indicates 8 substitutions for every ten positions. Bootstrap values (n=1000 bootstrap replicates) are displayed at branches. B) Full table of BLASTP results for each OTU. C) Counts of *Chloroflexus* and *Roseiflexus* OTUs (assigned by best BLASTP match) in each site, divided into temperature groups.

Figure S4. Ratio of Calvin cycle genes with temperature.

The ratio of *rbcS* genes (x axis) to both *rbcL* and *prk* genes (y axis) is shown. Points are shaded by site temperature.

Figure S5. Distribution and abundance of *rbcL* OTUs.

Rank abundance plots for each site are displayed in increasing temperature order. Plots display abundances as normalized *ln*(1 + reads mapped) for each *rbcL* OTU and OTUs are ranked in order from most to least abundant. Bars are labeled with the OTU number. Striped bars represent OTUs that are present in more than one site.

Figure S6. Type I reaction center and rTCA cycle genes.

A) The abundance (normalized *ln*(1 + reads mapped) of type I anoxygenic photosynthesis reaction center genes are shown as box plots for each site. Triangles represent the mean abundance for the gene set and dots represent individual gene abundances, shaded by reaction center gene. Boxes represent the inter quartile range (Q1-Q3) and whiskers (lines) represent the maximum and minimum, with outliers removed (+/-2.5 standard deviations from the mean). Sites are ordered by increasing temperature. B) rTCA cycle gene distribution with temperature. The abundance (normalized *ln*(1 + reads mapped) for genes in the rTCA cycle plotted as boxplots for each site. Triangles

represent the mean abundance for the gene set and dots represent individual gene abundances, shaded by gene. Boxes represent the inter quartile range (Q1-Q3) and whiskers (lines) represent the maximum and minimum, with outliers removed (+/-2.5 standard deviations from the mean). Sites are ordered by decreasing temperature.

Figure S7. Maximum likelihood-inferred NifH tree, OTU best BLAST match, and conserved region alighment.
A) Maximum likelihood-inferred phylogenetic reconstruction of translated *nifH* OTUs recovered from YNP sites (bold) aligned to all reference sequences in the Gaby & Buckley (2014) *nifH* database (1). NCBI taxonomy IDs are shown for each un-collapsed reference taxon. The tree is rooted with *Synechococcus* BchlL. The scale bar indicates 5 substitutions for every ten positions. Bootstrap values (n=1000 bootstrap replicates) are displayed at branches. Bootstrap values less than 50 are not shown. **Collapsed clades**: *Firmicute*s sp (*Desulfotomaculum* acetoxidans DSM 711, *Thermoanaerobacterium* aotearoense SCUT27); *Chlorobaciulum* sp & OTU10 (*Chlorobaculum parvum* & OTU10); *Cyanobacteria* sp (*Leptolyngbya* boryana dg5, *Oscillatoriales* sp. JSC-1, *Cyanobacterium* sp JSC-1); *Oscillilochloris* sp. (*Oscillochloris fontis*, *Oscillochloris trichoides* DG-6); *Desulfotomaculum & Desulfitobacterium* sp. (*Desulfotomaculum acetoxidans*, *Desulfitobacterium haniense* Y51). B) Full table of BLASTP results for each OTU. C) Alignment of conserved NifH metal binding site (35 amino acids) with 25 NifH OTUs. Arrows indicate metal binding position. Asterisks (*) indicate *Roseiflexus* NifH sequences.

Table S2. Kruskal-Wallace significance for photosynthesis genes.
Kruskal-Wallace significance values comparing photosynthesis gene (*psb, puf,* and *psc*) abundances across each site.

Table 3. Kruskal-Wallace significance for carbon fixation pathway genes.
Kruskal-Wallace significance values comparing carbon fixation pathway gene abundances in the 1) Calvin cycle (RuBisCO large and small subunits, phosphoribulokinase*,*), 2) 3-hydroxypropionate bicycle (*mcl, mcr, pccA*) and 3) the reverse tri-carbo-carboxylic acid cycle (ATP citrate lyase alpha and beta subunits, isocitrate dehydrogenase) across each site in the analysis.

Supplemental Material: "rbcL and psbA reference sequences" – reference sequences acquired from the Joint Genome Institute for *rbcL* and *psbA* OTU analysis.

Table S1. Sample site and metagenome meta-data.

A.

Site ID, Site Name (corresponds with Hamilton *et al.* 2019)		JGI Genome ID	Library name	pH	Temperature (ºC)	Sulfide (µM)	Fe^{2+} (µM)	SiO_2 (mM)	$\delta^{15}N$ (‰)	SO_4^{2-} (mM)	PO_4^{3-} (µM)	Mo (nM)
**RCA3**	Rabbit Creek OF1	3300028606	626A	9.14	68.40	0.34	bdl*	3.14	-0.93	0.20	5.43	290.61
**RCA4**	Rabbit Creek OF2	3300028609	626B	9.24	62.30	0.44	bdl	2.48	-1.32	0.19	5.40	287.30
**RCA5**	Smoking Gun Spring	3300028611	626C	9.44	62.40	19.00	bdl	4.19	-2.38	0.16	6.44	304.56
**RCA6**	Rabbit Creek OF3	3300028818	626D	9.29	62.30	0.25	bdl	3.43	-2.09	0.19	5.32	301.98
**BG1**	Boulder Geyser	3300028893	629B	8.68	68.50	45.53	0.90	2.13	4.09	0.19	3.18	483.06
**WCA1**	Mouthful Geyser	3300028816	629F	8.80	71.00	3.12	2.33	4.63	4.12	0.16	7.15	259.47
**WCA2**	Stumped Spring	3300028617	629H	8.56	69.40	1.25	0.36	3.96	4.26	0.15	9.07	267.31
**GCA3**	Mixy Fritzy	3300028820	630D	7.30	62.70	0.87	0.12	3.71	2.82	1.39	4.68	683.71

*bdl = below detection limit (< 0.01 µM)

B.

Site ID	NCBI Accession	JGI IMG genome ID	ORFs identified	Assembly reads	N50	# Contigs
**RCA3**	SRR14150299	3300028606	204899	796348	62364	796348
**RCA4**	SRR14150300	3300028609	332336	916781	66351	916781
**RCA5**	SRR14150301	3300028611	150190	375420	17478	375420
**RCA6**	SRR14150302	3300028818	312239	887224	61306	887224
**BG1**	SRR14150303	3300028893	353535	1122653	89147	1187870
**WCA1**	SRR14150304	3300028816	184233	1187870	40218	569186
**WCA2**	SRR14150305	3300028617	233562	569186	57513	788302
**GCA3**	SRR14150306	3300028820	286391	788302	29204	554619
		**Mean**	257173	830473	52948	759469
		**Standard deviation**	74524	267811	22845	254236

Figure S1. Maximum-likelihood inferred PsbA tree with OTU BLASTP results

A.

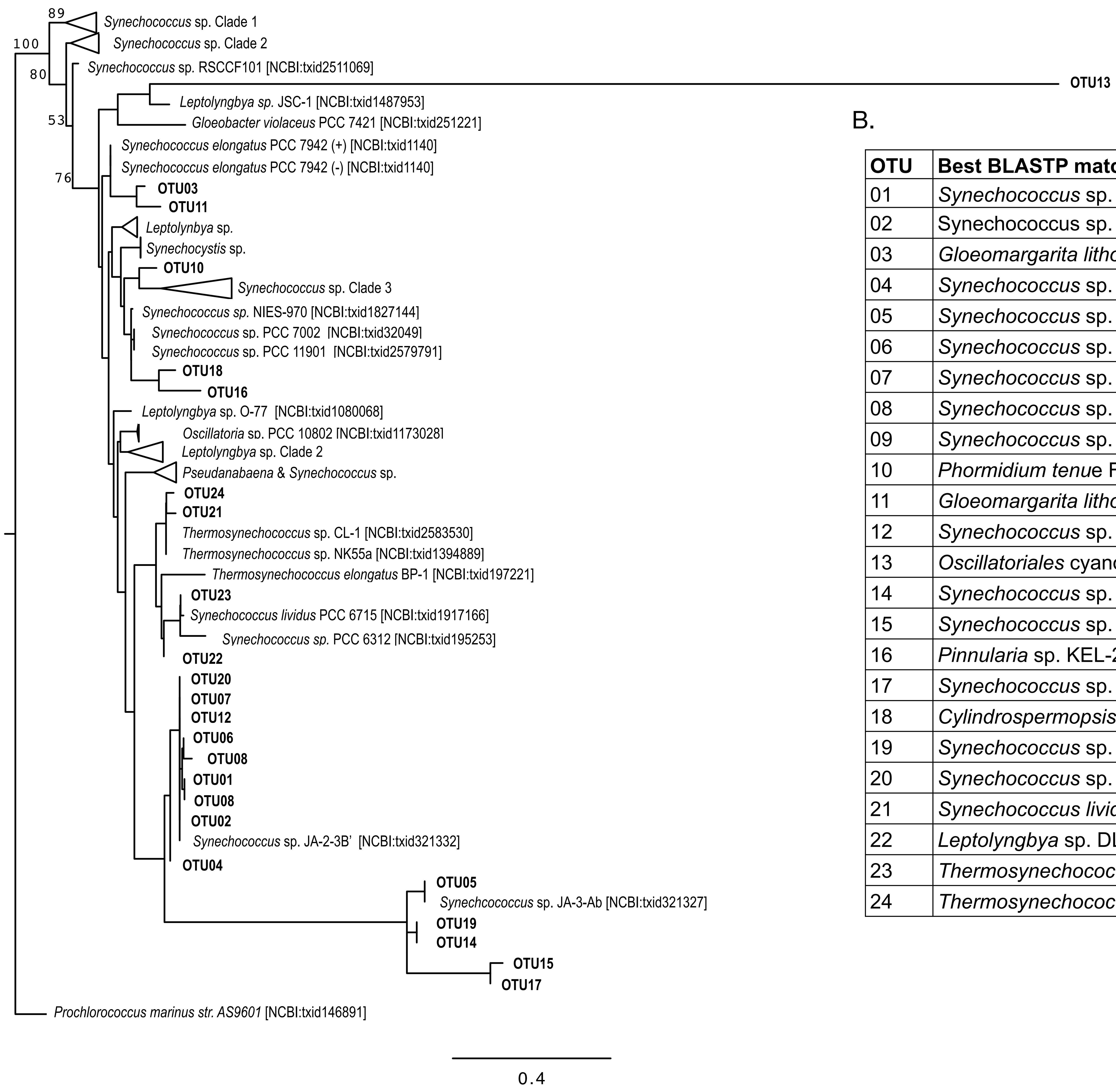

B.

OTU	Best BLASTP match	% Identity	E-Value	NCBI ID	Abundance
01	*Synechococcus* sp. 60AY4M2	100.00	9E-13	1353262	0.15
02	Synechococcus sp. 60AY4M2	96.67	1E-23	1353262	0.13
03	*Gloeomargarita lithophora*	96.00	2E-61	1188228	0.54
04	*Synechococcus* sp. OH28	98.11	6E-29	139350	0.57
05	*Synechococcus* sp. JA-2-3B'a(2-13)	100.00	2E-11	321332	0.68
06	*Synechococcus* sp. OH20	100.00	7E-82	139337	1.02
07	*Synechococcus* sp. OH2	100.00	1E-81	136798	0.94
08	*Synechococcus* sp. 63AY4M2	97.98	3E-166	1353266	1.23
09	*Synechococcus* sp. JA-2-3B’a(2-13)	99.59	9E-163	321332	1.69
10	*Phormidium tenue* FACHB-1052	96.60	3E-79	2692910	0.01
11	*Gloeomargarita lithophora*	94.44	2E-61	1188228	0.01
12	*Synechococcus* sp. OH2	100.00	3E-55	136798	0.37
13	*Oscillatoriales* cyanobacterium SM2_3_0	71.43	5E-06	2720468	0.01
14	*Synechococcus* sp. 63AY4M2	96.00	5E-07	1353266	0.13
15	*Synechococcus* sp. 63AY4M2	96.56	1E-44	1353266	0.01
16	*Pinnularia* sp. KEL-2015	100.00	3E-56	1689196	0.01
17	*Synechococcus* sp. 63AY4M2	97.00	5E-54	1353262	0.06
18	*Cylindrospermopsis raciborskii* S14	100.00	3E-39	2014883	0.01
19	*Synechococcus* sp. 63AY4M2	98.96	5E-117	1353266	1.18
20	*Synechococcus* sp. JA-2-3B'a(2-13)	100.00	5E-163	321332	0.04
21	*Synechococcus lividus*	98.68	1E-101	33070	0.02
22	*Leptolyngbya* sp. DLM2.Bin15	99.14	6E-79	2480212	0.01
23	*Thermosynechococcus* sp. M98_K2018_005	100.00	4E-42	2747811	0.01
24	*Thermosynechococcus* sp. M46_R2017_013	97.96	7E-28	2747807	0.01

Figure S2. Alpha diversity of *psbA, pufLM, nifH,* and *rbcL* gene variants across sites.

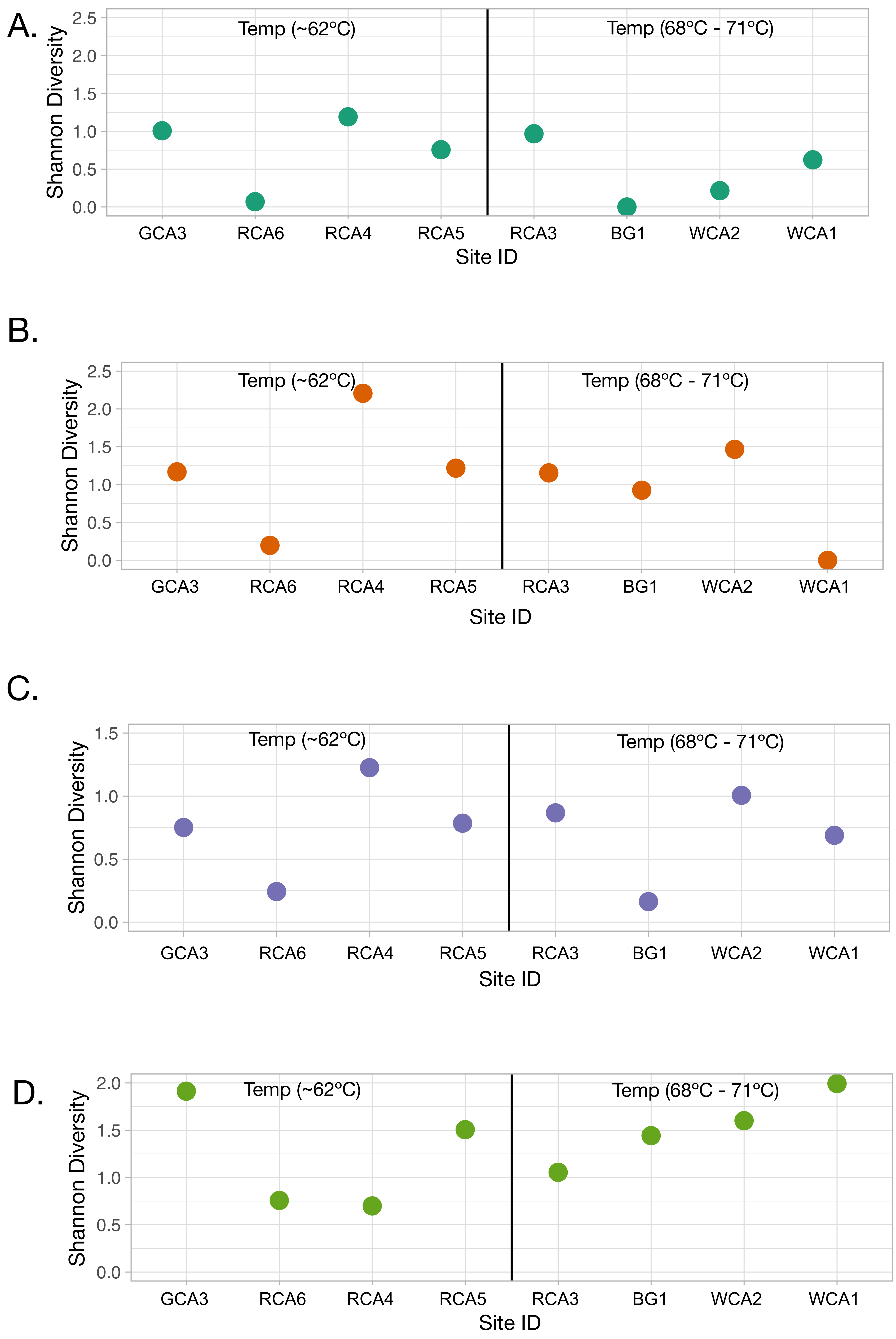

Figure S3. Maximum likelihood-inferred PufLM tree, OTU best BLASTP match, and PufLM BLASTP counts for *Roseiflexus* and *Chloroflexus* genera.

A.

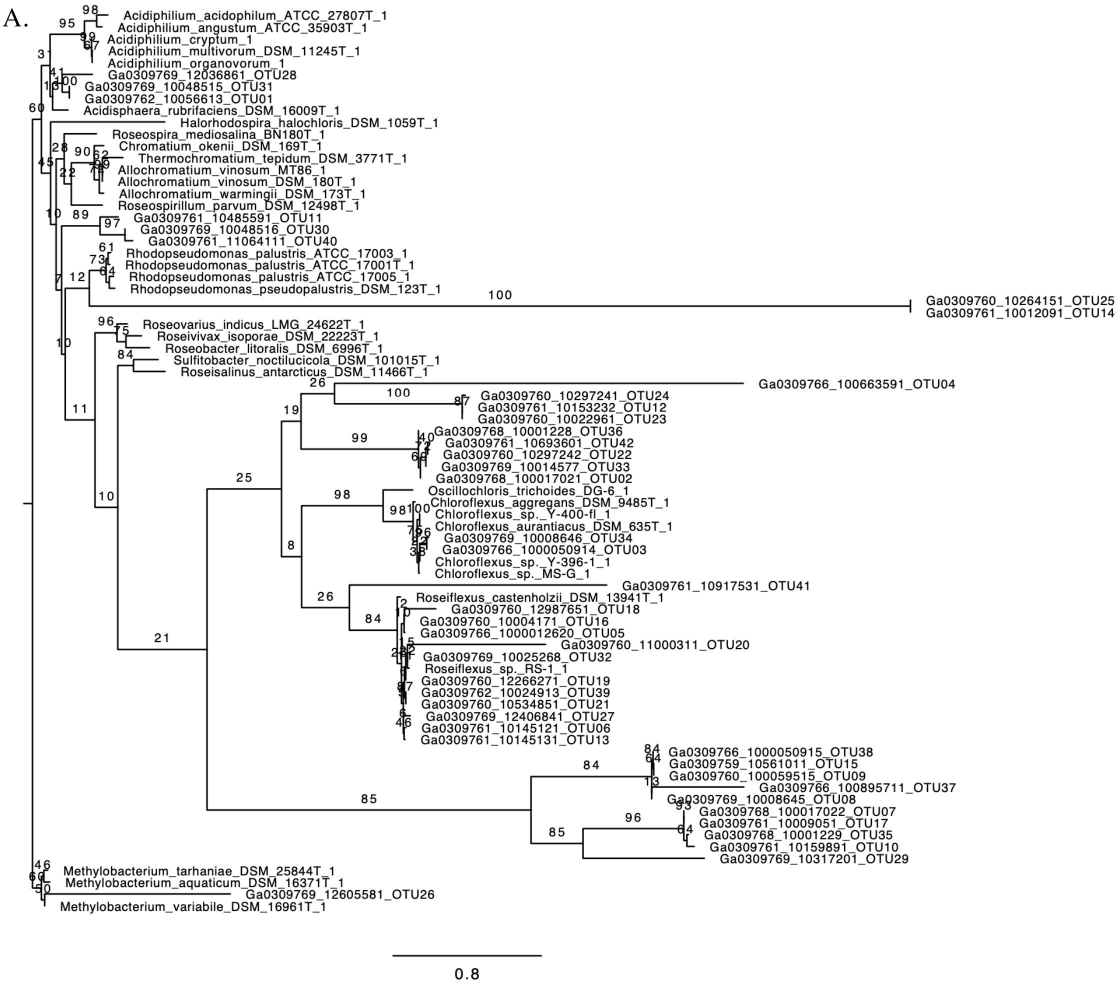

B.

Abundance	OTU	Best BLASTP match	% Identity	E-Value	NCBI ID
0.019	01	Actinobacteria bacterium	99.64	3E-165	1883427
0.406	02	Chloroflexi bacterium	99.60	0.0	2026724
0.525	03	*Chloroflexus* sp. Y-396-1	100.00	2E-61	867845
0.036	04	*Chloroflexus* sp. Y-396-1	100.00	3E-119	867845
0.515	05	*Roseiflexus* sp	100.00	0.0	2562120
0.339	06	*Roseiflexus* sp	97.70	0.0	2562120
0.604	07	Chloroflexi bacterium	100.00	0.0	2026724
0.617	08	*Chloroflexus* sp.	100.00	0.0	1904827
0.149	09	*Chloroflexus* sp.	100.00	0.0	1904827
0.238	10	*Chloroflexus* sp.	95.36	1E-123	1904827
0.017	11	Uncultured Gammaproteobacteria	85.07	6E-79	86473
0.01	12	Chloroflexi bacterium	100.00	1E-80	2026724
0.102	13	*Roseiflexus* sp	97.19	0.0	2562120
0.282	14	*Roseiflexus* castenholzii	100.00	5E-22	120962
0.020	15	*Chloroflexus* sp.	100.00	2E-90	1904827
0.80	16	*Roseiflexus* sp	99.68	0.0	2562120
0.01	17	Chloroflexi bacterium	100.00	3E-129	2026724
0.074	18	*Roseiflexus castenholzii*	100.00	3E-30	120962
0.097	19	*Roseiflexus castenholzii*	97.30	1E-45	120962
0.29898	20	*Roseiflexus* sp. RS-1	97.04	1E-65	357808
0.539	21	*Roseiflexus* sp	100	6E-123	2562120
0.300	22	Chloroflexi bacterium	96.20	2E-166	2026724
0.225	23	Candidatus Roseilinea sp. NK_OTU-006	97.44	3E-70	2704250
0.300	24	*Candidatus* Roseilinea sp. NK_OTU-006	96.58	5E-77	2704250
1.26	25	*Roseiflexus castenholzii*	100.00	1E-08	120962
0.01	26	*Roseitalea* sp.	77.43	1E-11	1915402
0.01	27	*Roseiflexus* castenholzii	98.55	2E-39	120962
0.01	28	Elioraea rosea	97.02	1E-34	2492390
0.034	29	*Roseiflexus* sp. RS-1	100.00	1E-40	357808
0.030	30	*Rubrivivax* sp.	86.75	0.0	50259
0.030	31	Actinobacteria bacterium	99.64	3E-165	1883427
0.054	32	*Roseiflexus* sp. RS-1	100.00	0.0	357808
0.511	33	*Candidatus* Roseilinea sp. NK_OTU-006	91.94	0.0	2704250
1.10	34	*Chloroflexus* sp.	100.00	0.0	1904827
0.339	35	Chloroflexi bacterium	97.64	0.0	2026724
0.339	36	Chloroflexi bacterium	100.00	1E-80	2026724
0.070	37	*Chloroflexus aurantiacus*	100.00	7E-83	1108
1.27	38	*Chloroflexus* sp.	100.00	0.0	1904827
0.801	39	*Roseiflexus* sp	100.00	0.0	2562120
0.01	40	Actinobacteria bacterium	99.64	8E-35	1883427
0.01	41	Betaproteobacterium	93.37	0.059	1891241
0.01	42	Chloroflexi bacterium	98.04	3E-57	2026724

C.

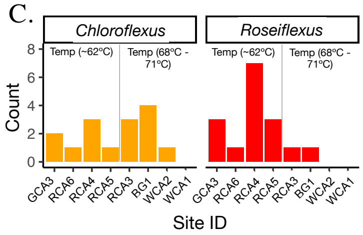

Figure S4. Ratio of key Calvin cycle genes with temperature.

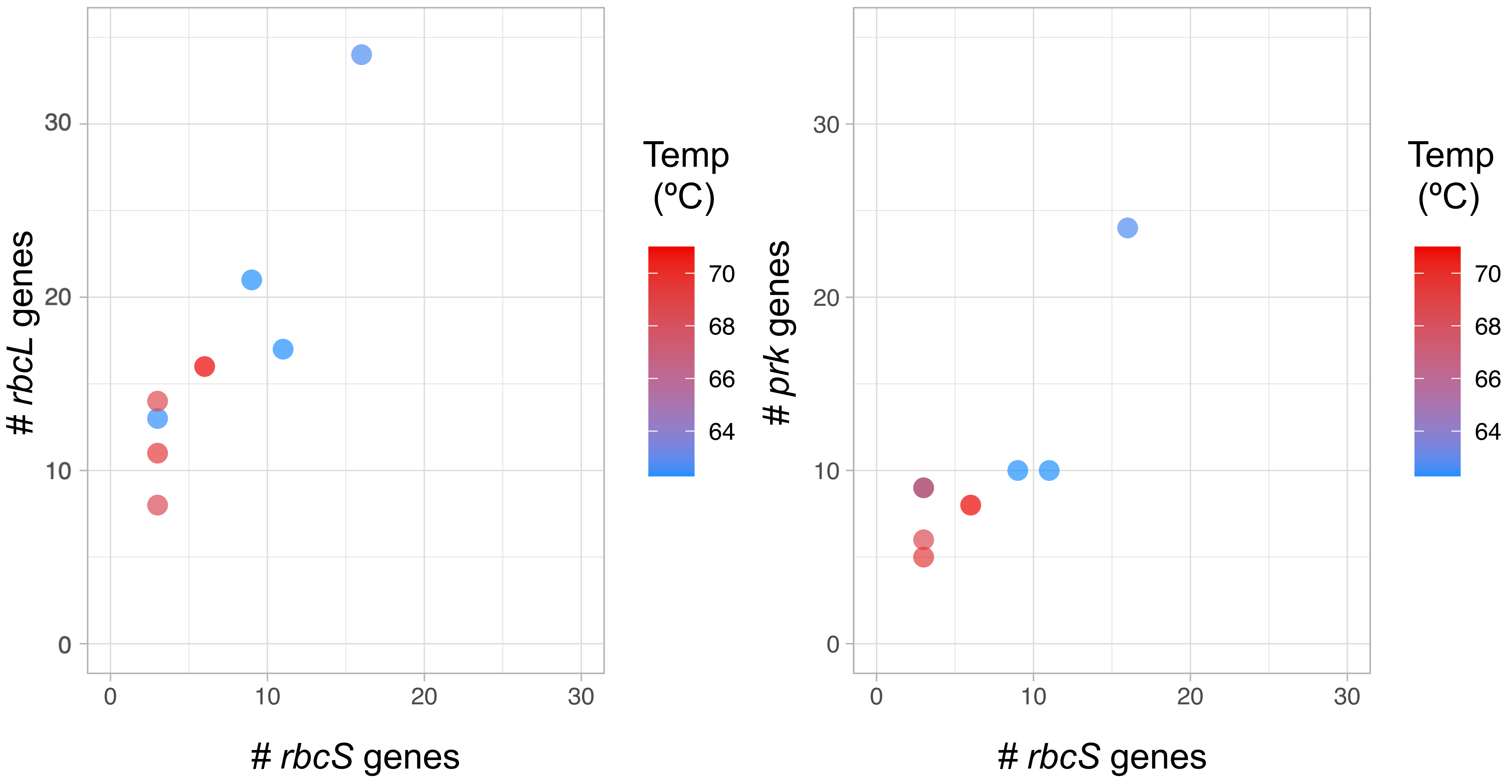

Figure S5. Richness and distribution of *rbcL* gene variants.

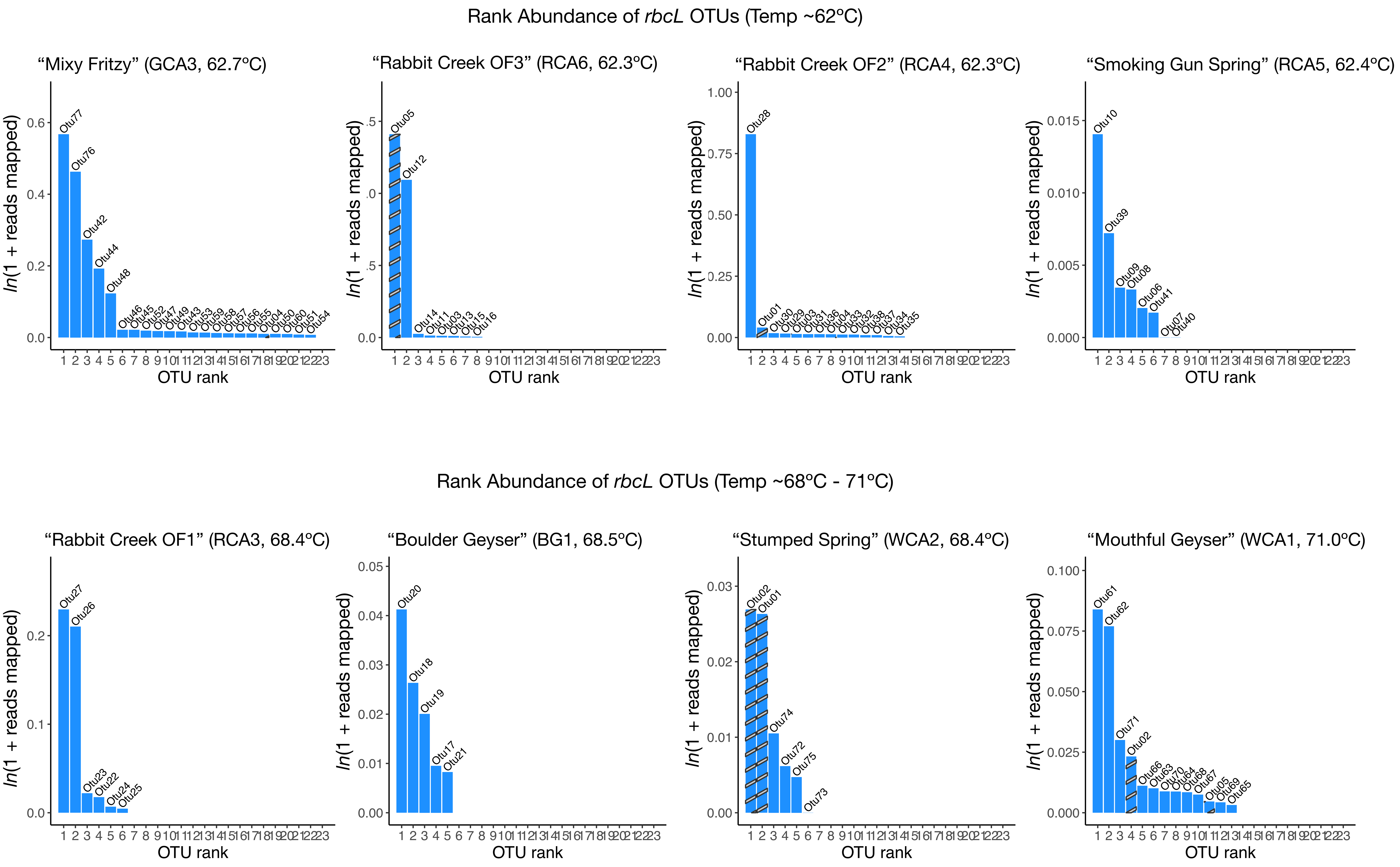

Figure S6. Abundance and distribution of type I reaction center genes and key genes in the rTCA cycle.

A. Type I reaction center genes.

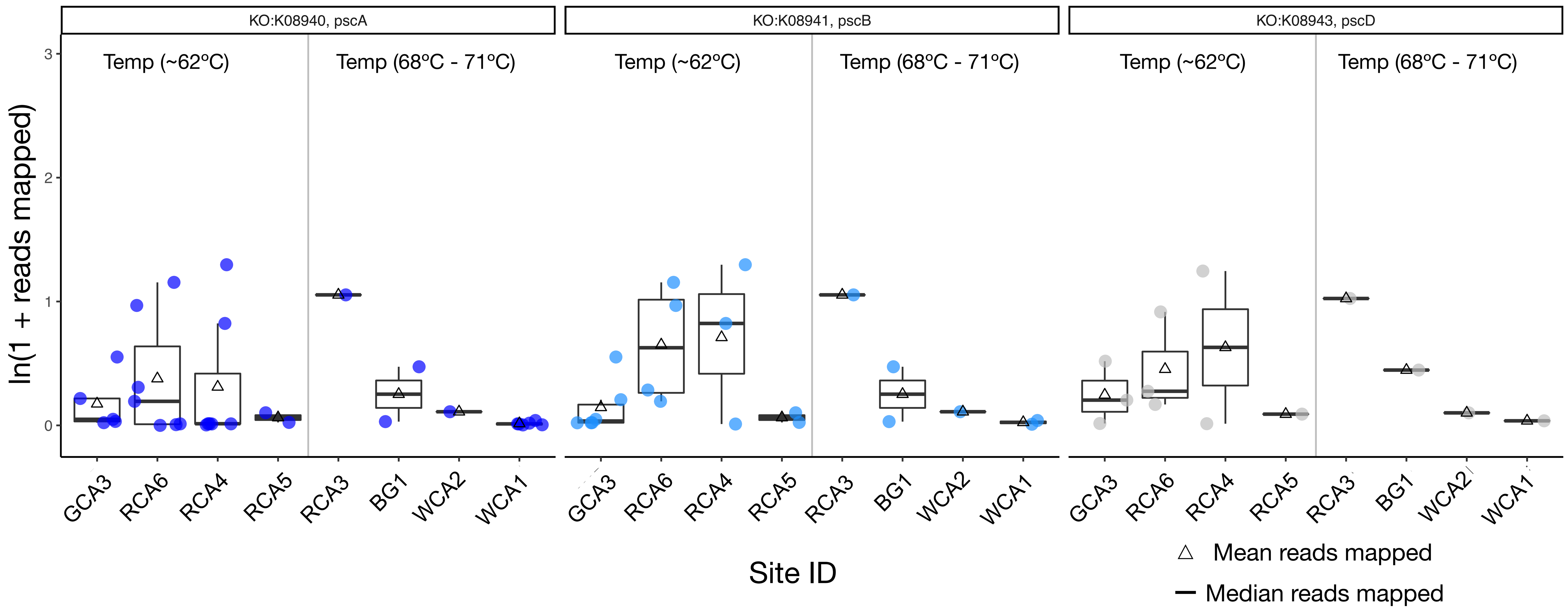

B. Key genes in the rTCA cycle.

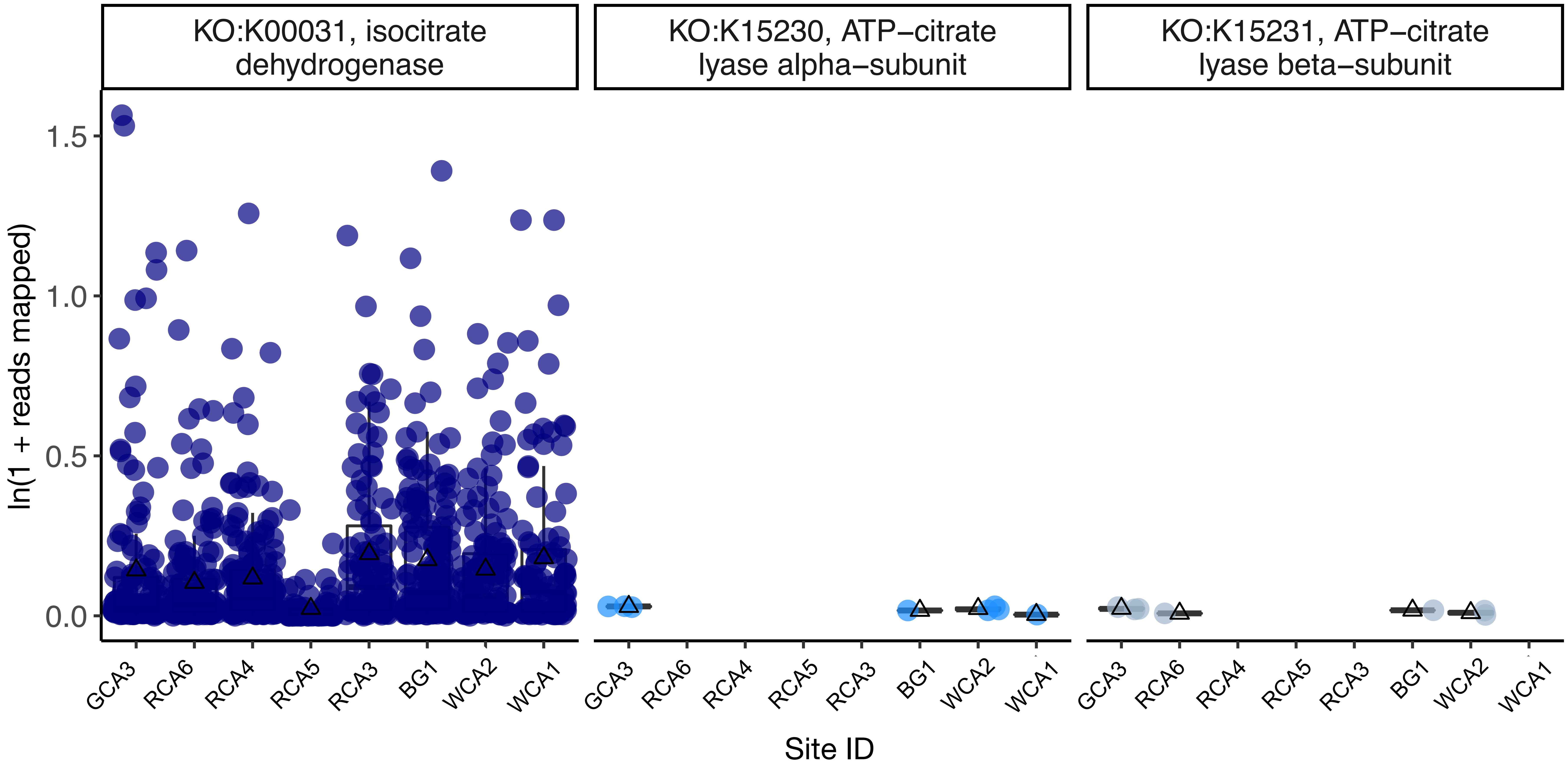

Figure S7. NifH maximum likelihood-inferred phylogenetic tree, OTU BLASTP results, and conserved region alignment.

A.

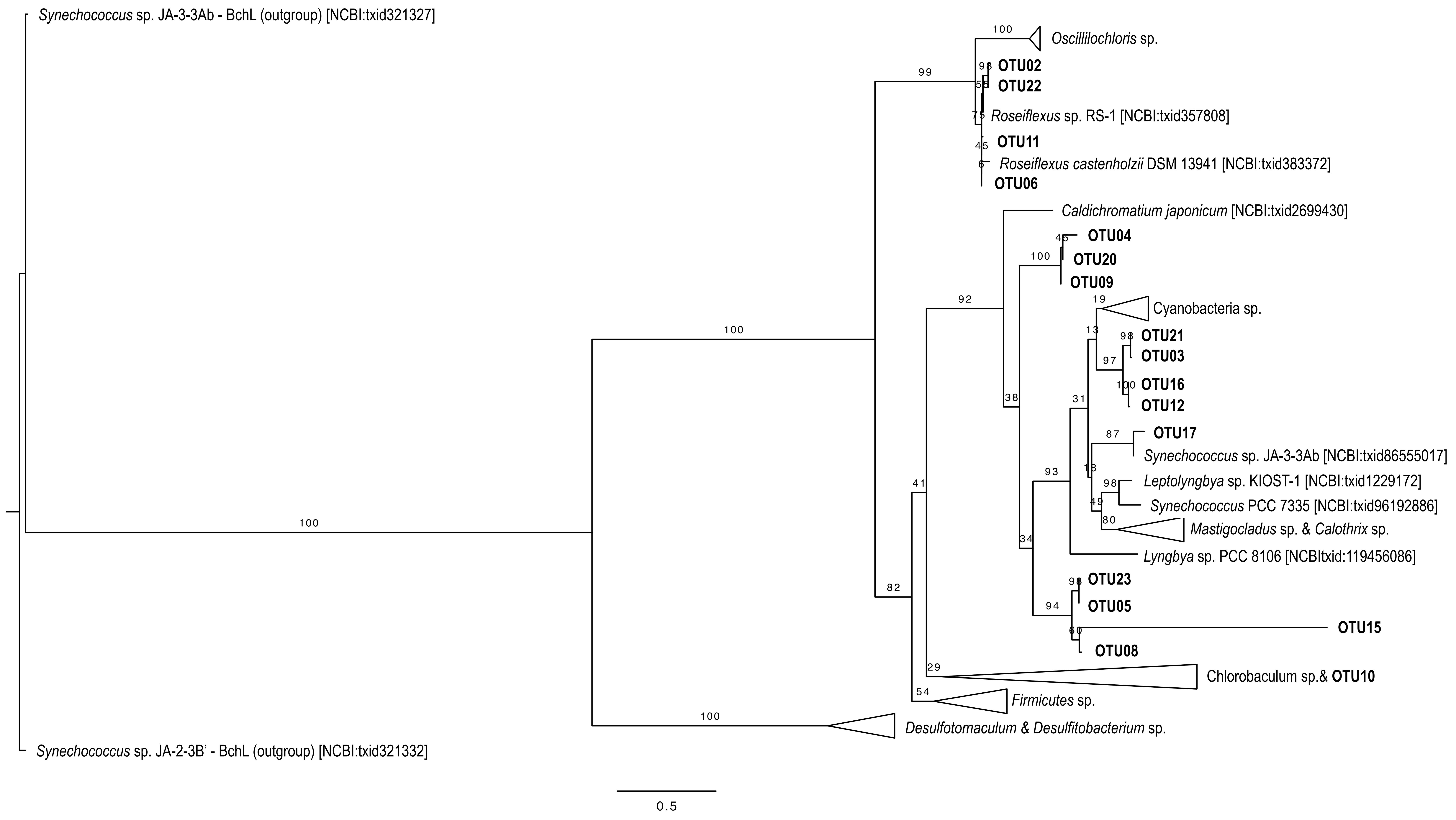

B.

OTU	Best BLASTP match	E-Value	% Identity	NCBI ID	Abundance
01	unclassified Synechococcus	0	100	2626047	1.74
09	*Hydrogenobacter thermophilus*	2E-166	95.34	940	1.71
02	*Roseiflexus* sp	0	100	2562120	0.779
03	*Synechococcales M55_K2018_004*	0	90.34	2774347	0.680
16	*Synechococcales* cyanobacterium M58_A2018_015	0	89.26	2774348	0.301
22	*Roseiflexus* sp	6E-180	99.63	2562120	0.2709
06	*Roseiflexus* sp	3E-180	100	2562120	0.267
12	*Synechococcales* cyanobacterium M58_A2018_015	0	88.93	2774348	0.087
08	Dissulfurispira thermophila	0	96.04	2715679	0.066
05	Dissulfurispira thermophila	2E-174	94.16	2715679	0.0415
11	*Roseiflexus* sp	4E-140	100	2562120	0.0415
15	Dissulfurispira thermophila	3E-101	71.48	2715679	0.028
07	*Synechococcales* cyanobacterium M58_A2018_015	1E-43	91.46	2774348	0.0249
25	*Nitrospirae* bacterium	4E-22	90.00	2026887	0.0127
04	*Hydrogenobacter thermophilus*	3E-107	95.72	940	0.0115
26	*Hydrogenobacter thermophilus*	5E-54	98.72	940	0.01
10	*Methanothermobacter thermautotrophicus*	2E-23	98.59	145262	0.01
13	*Uncultured bacterium*	1E-26	88.41	77133	0.01
14	Dissulfurispira thermophila	6E-39	93.15	2715679	0.01
17	*Uncultured bacterium*	2E-46	97.30	77133	0.01
18	*Hydrogenobacter thermophilus*	1E-47	91.95	940	0.01
19	unclassified Synechococcus	7E-13	100	2626047	0.01
20	*Uncultured bacterium*	3E-29	94.52	77133	0.01
21	*Leptolyngbya sp. 'hensonii'*	0	90.91	1922337	0.01
23	*Thermodesulfovibrio aggregans*	1E-60	93.00	86166	0.01
24	*Mycobacterium avium*	0.037	78.26	1764	0.01

C.

```
NifH metal binding site        --------CAGRGVITAINFLEENGAYE-NVDYVSYDVLGDVVC----------------  35
Ga0309760_10424241_OTU15       GGPEPGVGCAGRGVITAINFLEENGAYGDDLDFVFYDVLGDVVCGGFAMPIREGKAKEIY  30
Ga0309766_100053511_OTU09      GGPEPGVGCAGRGVITAINFLEENGAFDDDLDYVFYDVLGDVVCGGFAMPIREGKAQEIY  30
Ga0309767_10288151_OTU04       GGPEPGVGCAGRGVITAINFLEENGAFDDDLDYVFYDVLGDVVCGGFAMPIREGKAQEIY  31
Ga0309761_11106571_OTU20       --PEPGVGCAGRGVITAINFLEENGAFDDDLDYVFYDVLGDVVCGGFAMPIREGKAQEIY  30
Ga0309759_100008916_OTU02*     GGPEPGVGCGGRGVITAIQTLETLGAYKDDLDYVFYDVLGDVVCGGFAMPIREGYAEEIY  26
Ga0309761_10009524_OTU22*      GGPEPGVGCGGRGVITAIQTLETLGAYKDDLDYVFYDVLGDVVCGGFAMPIREGYAEEIY  24
Ga0309761_100056118_OTU06*     GGPEPGVGCGGRGVITAIQTLETLGAYKDDLDYVFYDVLGDVVCGGFAMPIREGYAEEIY  24
Ga0309769_10025703_OTU11       GGPEPGVGCGGRGVITAIQTLEALGAYKDDLDYVFYDVLGDVVCGGFAMPIREGYAEEIY  24
Ga0309767_11196241_OTU17       GGPEPGVGCAGRGIITAINFLEENGAYE-DLDFVCYDVLGDVVCGGFAMPIREGKAQEIY  30
Ga0309759_100024911_OTU01      GGPEPGVGCAGRGIITAINFLEENGAYE-DLDFVCYDVLGDVVCGGFAMPIREGKAQEIY  30
Ga0309760_100039210_OTU03      GGPEPGVGCAGRGIITAINFLEENGAYE-DLDFVSYDVLGDVVCGGFAMPIREGKAQEIY  30
Ga0309761_10069682_OTU21       GGPEPGVGCAGRGIITAINFLEENGAYE-DLDFVSYDVLGDVVCGGFAMPIREGKAQEIY  30
Ga0309762_100055611_OTU16      GGPEPGVGCAGRGIITAINFLEENGAYE-DIDFVSYDVLGDVVCGGFAMPIREGKAQEIY  30
Ga0309769_100078411_OTU12      GGPEPGVGCAGRGIITAINFLEENGAYE-DIDFVSYDVLGDVVCGGFAMPIREGKAQEIY  30
Ga0309769_12300751_OTU10       ----------------AITLMERYGVYEKDLDFVFFDVLGDVVCGGFAMPVRDGKAEEIY  10
Ga0309766_100121752_OTU08      GGPEPGVGCAGRGVITAINFLEENGAYEADIDFVFYDVLGDVVCGGFAMPIREGKAKEIY  28
Ga0309760_11486201_OTU23       GGPEPGVGCAGRGVITAINFLEENGAYDADLNFVFYDVLGDVVCGGFAMPIREGKAKEIY  29
Ga0309761_10026544_OTU05       GGPEPGVGCAGRGVITAINFLEENGAYDADLNFVFYDVLGDVVCGGFAMPIREGKAKEIY  28
```

Table S2. Kruskal-Wallace significance for photosynthesis genes.

Reaction center comparison	KO.K08943..pscD. Comparison	KO.K08943..pscD. Z	KO.K08943..pscD. P.unadj	KO.K08943..pscD. P.adj	KO.K13992..pufC. Comparison	KO.K13992..pufC. Z	KO.K13992..pufC.P .unadj	KO.K13992..pufC.P .adj	KO.K08929..pufM. Comparison	KO.K08929..pufM. Z	KO.K08929..pufM. P.unadj	KO.K08929..pufM. P.adj	KO.K08941..pscB. Comparison	KO.K08941..pscB. Z	KO.K08941..pscB. P.unadj	KO.K08941..pscB. P.adj
1	RCA3 - RCA4	1.04828483672192	0.29450739368011	1	RCA3 - RCA4	-0.295220022940142	0.767825807195903	0.977232845522059	RCA3 - RCA4	-1.18056803697977	0.237774367410498	0.739742476388216	RCA3 - RCA4	0.7909115788387	0.4289955911145	0.800791770080399
2	RCA3 - RCA5	1.45254607840513	0.146349826662573	1	RCA3 - RCA5	0.876175252577526	0.380934767859793	1	RCA3 - RCA5	1.41044718967113	0.158407679703885	0.63363071881554	RCA3 - RCA5	1.44749372891149	0.147758691557173	0.591034766228691
3	RCA4 - RCA5	0.628970902033151	0.529368106184798	0.926394185823396	RCA4 - RCA5	1.65618633651909	0.0976840893831072	1	RCA4 - RCA5	3.47187853968158	0.000516830114438465	0.014471243204277	RCA4 - RCA5	0.9415838181384	0.346405757692238	0.881760110489332
4	RCA3 - RCA6	0.815374248327211	0.414858120146241	1	RCA3 - RCA6	-0.111143786045242	0.911502327566289	0.981617891225234	RCA3 - RCA6	-0.844090841376329	0.398618645170867	0.858563235752637	RCA3 - RCA6	0.432449982093868	0.665411439778476 9	0.84689105172607
5	RCA4 - RCA6	-0.375045784750797	0.707626416361264	0.900615439005245	RCA4 - RCA6	0.287577955400223	0.773669821386574	0.94185891299235	RCA4 - RCA6	0.213012544362991	0.831317178234244	0.931075239622353	RCA4 - RCA6	-0.562703958762664	0.573636488112231	0.845359035112762
6	RCA5 - RCA6	-0.963624111659432	0.33523441379596	1	RCA5 - RCA6	-1.4400024240445	0.149866713259349	1	RCA5 - RCA6	-2.52378046940363	0.011610040599905	0.108360378932447	RCA5 - RCA6	-1.48877473663755	0.136546697013435	0.637217919396028
7	RCA3 - BG1	0.544704779401922	0.585956577194652	0.911488008969459	RCA3 - BG1	-0.0550019098216927	0.956136942280222	0.956136942280222	RCA3 - BG1	-1.06938199120987	0.284897578726376	0.725193836758049	RCA3 - BG1	1.05272271193563	0.29246809625552	0.909900743906062
8	RCA4 - BG1	-0.419313934688767	0.674986713970489	0.899982285293986	RCA4 - BG1	0.270253982183343	0.786964865380356	0.918125676277083	RCA4 - BG1	-0.0665664201134347	0.946926879295426	0.981998245195256	RCA4 - BG1	0.41194292043555	0.68038126079271	0.828290230530256
9	RCA5 - BG1	-0.907841299003204	0.363962075102477	1	RCA5 - BG1	-1.07253724152301	0.283478795970575	1	RCA5 - BG1	-2.79662160123105	0.00516399742383566	0.0722959639336992	RCA5 - BG1	-0.483493778415228	0.628745176273833	0.838326901698444
10	RCA6 - BG1	-0.14824986333222	0.882145581296006	0.882145581296006	RCA6 - BG1	0.0556234200555013	0.955641806879004	0.991035947874522	RCA6 - BG1	-0.236287351359844	0.813209701242479	0.948744651449559	RCA6 - BG1	0.930484210398471	0.352120435634723	0.821614349814353
11	RCA3 - WCA1	1.63411433820577	0.102234844735488	1	RCA3 - WCA1	-0.552306089778777	0.580738659001765	1	RCA3 - WCA1	-0.423547361687055	0.671895951026881	0.940654331437633	RCA3 - WCA1	1.90805991538333	0.0563834727847159	0.526245745990682
12	RCA4 - WCA1	0.838627869377535	0.401678166469773	1	RCA4 - WCA1	-0.393626697253523	0.693856667725117	1	RCA4 - WCA1	0.734893278052319	0.462404492491704	0.809207861860482	RCA4 - WCA1	1.55949819879172	0.118878510557526	0.665719659122148
13	RCA5 - WCA1	0.181568259800641	0.855921559768773	0.887622358278727	RCA5 - WCA1	-1.53630729219073	0.124463031870288	1	RCA5 - WCA1	-2.01447702332578	0.0439594748218096	0.246173059002134	RCA5 - WCA1	0.564076074817766	0.57270235977831	0.890870337432927
14	RCA6 - WCA1	1.18599890665776	0.235622731299505	1	RCA6 - WCA1	-0.587474297667709	0.556885207496872	1	RCA6 - WCA1	0.441069722538375	0.659162522082017	1	RCA6 - WCA1	2.14011368391648	0.0323455804838596	0.905676253548068
15	BG1 - WCA1	1.08940955880384	0.275973318013738	1	BG1 - WCA1	-0.550019098216927	0.582306274413217	1	BG1 - WCA1	0.677357073898219	0.498179426941981	0.820530820845616	BG1 - WCA1	1.04756985323299	0.294836833019914	0.82554313245576
16	RCA3 - WCA2	1.27097781860449	0.203736539889507	1	RCA3 - WCA2	0.351467511677404	0.725237641653548	1	RCA3 - WCA2	-0.753974381442913	0.45086460496418	0.841613929266469	RCA3 - WCA2	0.911684611677104	0.361934766921192	0.779551805676414
17	RCA4 - WCA2	0.419313934688767	0.674986713970489	0.944981399558685	RCA4 - WCA2	0.733577026699747	0.463206566654283	1	RCA4 - WCA2	0.324844130153561	0.745299043163196	0.90732057428563	RCA4 - WCA2	0.325669473639465	0.744674442644921	0.801957092079145
18	RCA5 - WCA2	-0.181568259800641	0.855921559768773	0.921761679750986	RCA5 - WCA2	-0.456091227369123	0.648324378274335	1	RCA5 - WCA2	-2.41464401667266	0.0157505949492622	0.110254164644835	RCA5 - WCA2	-0.394771016975861	0.693011886370233	0.776173312734661
19	RCA6 - WCA2	0.741249316661101	0.458542286255114	1	RCA6 - WCA2	0.555718930226211	0.578403013496116	1	RCA6 - WCA2	0.0945149405439376	0.924700114734885	0.995830892791414	RCA6 - WCA2	0.720749970156447	0.471063361733219	0.775869066384125
20	BG1 - WCA2	0.726273039202563	0.467671403904174	0.935342807808348	BG1 - WCA2	0.440015278573541	0.659926041549517	1	BG1 - WCA2	0.330802291903782	0.740793829577928	0.942828510371909	BG1 - WCA2	0	1	1
21	WCA1 - WCA2	-0.363136519601281	0.716502905261313	0.872264406405077	WCA1 - WCA2	0.903773601456181	0.366115462382092	1	WCA1 - WCA2	-0.346554781994438	0.728925821629132	0.971901095505509	WCA1 - WCA2	-0.8553372034477	0.392364497147589	0.784728994295177
22	RCA3 - GCA3	1.26012383832387	0.207624692002837	1	RCA3 - GCA3	0.636127238172725	0.524693493356249	1	RCA3 - GCA3	-0.0370911129132368	0.970412358022015	0.970412358022015	RCA3 - GCA3	1.59156714016638	0.111481998341443	0.780373988390101
23	RCA4 - GCA3	0.187522892375398	0.851250676521134	0.95340075770367	RCA4 - GCA3	1.31318916623407	0.18911921366613	1	RCA4 - GCA3	1.3400567540512	0.180226894314659	0.630794130101306	RCA4 - GCA3	1.13960576459638	0.254450582646337	0.890577039262178
24	RCA5 - GCA3	-0.518874521662771	0.603848246067344	0.889881625783454	RCA5 - GCA3	-0.317553674414978	0.750823518899001	1	RCA5 - GCA3	-1.69117672781872	0.0908030543691725	0.423747587056139	RCA5 - GCA3	-0.065795169495977	0.947540902088477	0.982635009573235
25	RCA6 - GCA3	0.628970902033151	0.529368106184798	0.988153798211622	RCA6 - GCA3	1.08770825302444	0.276723923176287	1	RCA6 - GCA3	0.907261478973441	0.364268539516693	0.849959925538951	RCA6 - GCA3	1.9141758707259	0.0555976877629052	0.778367628680673
26	BG1 - GCA3	0.592999453328881	0.553181518108311	0.911122500413688	BG1 - GCA3	0.797527692414544	0.425144626432893	1	BG1 - GCA3	1.15986328286396	0.246104473911002	0.689092526950806	BG1 - GCA3	0.526361355967815	0.598637160237304	0.838092024332225
27	WCA1 - GCA3	-0.741249316661101	0.458542286255114	0.9876295396264	WCA1 - GCA3	1.29625927778593	0.194886173177228	1	WCA1 - GCA3	0.435738111711142	0.663026750793016	0.977092053800234	WCA1 - GCA3	-0.756644449203735	0.449262903924975	0.786210081868706
28	WCA2 - GCA3	-0.29649972666444	0.766848475677745	0.894656554957369	WCA2 - GCA3	0.216043212964322	0.828954063781033	0.928428551434757	WCA2 - GCA3	0.806220757417234	0.420115572061853	0.840231144123706	WCA2 - GCA3	0.397891785041595	0.690709955770514	0.805828281732267

Reaction center comparison	KO.K08940..pscA. Comparison	KO.K08940..pscA. Z	KO.K08940..pscA. P.unadj	KO.K08940..pscA. P.adj	KO.K02703..psbA. Comparison	KO.K02703..psbA. Z	KO.K02703..psbA. P.unadj	KO.K02703..psbA. P.adj	KO.K08928..pufL. Comparison	KO.K08928..pufL.Z	KO.K08928..pufL.P .unadj	KO.K08928..pufL.P .adj	KO.K02706..psbD. Comparison	KO.K02706..psbD. Z	KO.K02706..psbD. P.unadj	KO.K02706..psbD. P.adj
1	RCA3 - RCA4	1.54322792203707	0.122775484820202	1	RCA3 - RCA4	0.82570966152506	0.408968810305318	0.636173704919383	RCA3 - RCA4	0.912870929175277	0.361310428526179	0.919699272612091	RCA3 - RCA4	0.209095499774709	0.834373687721087	0.934498530247617
2	RCA3 - RCA5	1.03273048665396	0.30173004229747	0.844844118432915	RCA3 - RCA5	3.07109474691169	0.00213275462015364	0.059717129364302	RCA3 - RCA5	2.19469056285087	0.0281858021479073	0.789202460141406	RCA3 - RCA5	2.48302109654301	0.0130273372117612	0.0911913604823286
3	RCA4 - RCA5	-0.480115353522643	0.631145371444249	0.883603520021948	RCA4 - RCA5	2.65457026560217	0.00794094993247017	0.0741155327030549	RCA4 - RCA5	1.55134350376268	0.120819386535671	0.676588564599756	RCA4 - RCA5	3.00523545620049	0.0026537533067004	0.0743050925876111
4	RCA3 - RCA6	1.24927974641096	0.211562773105067	0.987292941156978	RCA3 - RCA6	1.07794769868491	0.281057097247832	0.605353747918407	RCA3 - RCA6	0.639009650422694	0.522816653919089	0.914929144358405	RCA3 - RCA6	0.151244248370432	0.879783046584986	0.947458665553061
5	RCA4 - RCA6	-0.587896351252216	0.556601865707367	0.916756014106251	RCA4 - RCA6	0.316859193532632	0.751350446575564	0.809146634773684	RCA4 - RCA6	-0.335410196624968	0.737315677216418	0.938401771002714	RCA4 - RCA6	-0.076273739021727	0.939201318192751	0.939201318192751
6	RCA5 - RCA6	0.0881844526878322	0.929730074130419	0.964164521320435	RCA5 - RCA6	-2.30550587917588	0.0211382578686055	0.118374244064191	RCA5 - RCA6	-1.91876696518016	0.055013832353979	0.513462435303804	RCA5 - RCA6	-2.9840753879833	0.0028443668133353	0.0398211353866943
7	RCA3 - BG1	0.763322533613793	0.44527106666827	0.890542133336539	RCA3 - BG1	0.46702086635914	0.640484949192593	0.717343143095704	RCA3 - BG1	0.474341649025257	0.635256295997248	0.936167173048576	RCA3 - BG1	-0.897325892757372	0.369545043818887	0.646703826683052
8	RCA4 - BG1	-0.891642799399193	0.372584421989817	0.802489524285759	RCA4 - BG1	-0.245074962027407	0.806398389256724	0.836264996266233	RCA4 - BG1	-0.365148371670111	0.715000654688089	0.953334206250785	RCA4 - BG1	-1.18051030526204	0.237797314222203	0.512178830632437
9	RCA5 - BG1	-0.329956008798045	0.741433202171676	0.943642257309406	RCA5 - BG1	-2.39559991892779	0.0165931909563514	0.11615233669446	RCA5 - BG1	-1.61374306091976	0.106583169574888	0.746082187024213	RCA5 - BG1	-2.91475837138571	0.00355964024084349	0.0332233089145392
10	RCA6 - BG1	-0.499711898564383	0.617277953113152	0.909672773008856	RCA6 - BG1	-0.491918967943282	0.622776628690832	0.726572733472637	RCA6 - BG1	-0.0912870929175277	0.927264473525232	0.961607602174315	RCA6 - BG1	-1.12580688211741	0.260247270497764	0.485794904929159
11	RCA3 - WCA1	2.01956159407384	0.0434288822742174	1	RCA3 - WCA1	1.41004376958433	0.158526759153682	0.36989577135859	RCA3 - WCA1	0.790569415042095	0.429195300440349	0.92442064710229	RCA3 - WCA1	-0.112165736594672	0.910691995927047	0.94442132910953
12	RCA4 - WCA1	0.955508387150857	0.339320665980074	0.863725331585642	RCA4 - WCA1	0.80763339759032	0.419301654846473	0.617918228194802	RCA4 - WCA1	0	1	1	RCA4 - WCA1	-0.285289990438326	0.775421980832387	0.943991976665514
13	RCA5 - WCA1	1.12253313766734	0.261635826112966	0.915725391395381	RCA5 - WCA1	-1.41558177027551	0.156897991792993	0.399376706382163	RCA5 - WCA1	-1.22644472629902	0.220031363581412	0.770109772534941	RCA5 - WCA1	-2.0819702652755	0.0373451858117461	0.149380743246985
14	RCA6 - WCA1	1.52034093127944	0.128425311826805	0.719181746230109	RCA6 - WCA1	0.54111086473761	0.588431170002299	0.716350989568016	RCA6 - WCA1	0.273861278752583	0.784191229401619	0.878294176929813	RCA6 - WCA1	-0.241244331882302	0.809365753455317	0.94426004569787
15	BG1 - WCA1	1.52664506722759	0.126849294015145	0.887945058106017	BG1 - WCA1	0.89463007796884	0.370984867704687	0.611033899748896	BG1 - WCA1	0.316227766016838	0.751829634045849	0.877134573053491	BG1 - WCA1	0.679968641276256	0.496524317017907	0.631940039840972
16	RCA3 - WCA2	0.622171016838255	0.53382942174414	0.934201488052244	RCA3 - WCA2	1.54320711612758	0.122780531272839	0.381983875071054	RCA3 - WCA2	0.158113883008419	0.874367061162892	0.94162606586773	RCA3 - WCA2	1.63147353431147	0.102790439822139	0.28781323150199
17	RCA4 - WCA2	-0.720173030283964	0.471418467012896	0.879981138424072	RCA4 - WCA2	0.924122731648686	0.355422419323133	0.621989233815483	RCA4 - WCA2	-0.730296743340221	0.465208818452142	0.930417636904283	RCA4 - WCA2	1.85253953266638	0.0639483862443421	0.198950534982398
18	RCA5 - WCA2	-0.314309278546856	0.753286151801831	0.917044010889186	RCA5 - WCA2	-1.45926748233385	0.144491501690927	0.404576204734594	RCA5 - WCA2	-2.0010413955405	0.0453879290387944	0.635431006543122	RCA5 - WCA2	-0.839524794059828	0.40117488178459	0.591205088945712
19	RCA6 - WCA2	-0.426224854657856	0.669944008199781	0.893258677599708	RCA6 - WCA2	0.63408688157936	0.526024123534644	0.669485248135001	RCA6 - WCA2	-0.456435464587638	0.648076868139146	0.907307615394805	RCA6 - WCA2	1.86539288668347	0.0621263537051425	0.217442237967999
20	BG1 - WCA2	-0.0449013255066937	0.96418596031032	0.96418596031032	BG1 - WCA2	0.987928755759719	0.32318754118291	0.603283410208099	BG1 - WCA2	-0.316227766016838	0.751829634045849	0.915270858838425	BG1 - WCA2	2.236909469929	0.0252922575004449	0.141636642002491
21	WCA1 - WCA2	-1.20494851411128	0.228223178332706	0.912892713330823	WCA1 - WCA2	0.0449058525345327	0.964182351904408	0.964182351904408	WCA1 - WCA2	-0.632455532033676	0.527089256865538	0.868147011307945	WCA1 - WCA2	1.42419149642538	0.154391045811853	0.360245773560991
22	RCA3 - GCA3	0.923702228237874	0.355641371667758	0.829829867224768	RCA3 - GCA3	2.69136686867717	0.00711598936019222	0.0996238510426911	RCA3 - GCA3	1.5	0.133614402537716	0.623533878509342	RCA3 - GCA3	0.799076618832981	0.424245986187931	0.593944380663103
23	RCA4 - GCA3	-1.08944718150552	0.275956734829293	0.858532063913356	RCA4 - GCA3	2.20405522786278	0.0275204603128373	0.128428814793241	RCA4 - GCA3	0.645497224367903	0.518605016428726	0.968062697333621	RCA4 - GCA3	0.839673073203652	0.401091715497154	0.623920446328906
24	RCA5 - GCA3	-0.302352527178531	0.762383339948611	0.889447229940046	RCA5 - GCA3	-1.00420183609357	0.315281337298964	0.630562674597928	RCA5 - GCA3	-1.12228508389081	0.261741246190357	0.814306099258888	RCA5 - GCA3	-2.18831909771089	0.0286463667337149	0.133683044757336
25	RCA6 - GCA3	-0.552773693079156	0.580418384398531	0.902873042397714	RCA6 - GCA3	1.76528413298694	0.0775160248457401	0.27130608696009	RCA6 - GCA3	1.03279555898864	0.301699582478348	0.844758830939374	RCA6 - GCA3	0.879846228349668	0.378942617589469	0.624140781912066
26	BG1 - GCA3	0.0920203343586832	0.926681883654044	0.997965105473585	BG1 - GCA3	1.90625344009091	0.0566173318345371	0.226469327338148	BG1 - GCA3	0.9	0.368120250693519	0.858947251618211	BG1 - GCA3	1.61354569573334	0.106626003923182	0.271411646349917
27	WCA1 - GCA3	-1.93138342528973	0.0534356533405842	0.748099146768178	WCA1 - GCA3	0.774625956220689	0.438560715112361	0.584747620149815	WCA1 - GCA3	0.5	0.617075077451974	0.959894564925293	WCA1 - GCA3	0.735709954531263	0.461907230302352	0.615876307069803
28	WCA2 - GCA3	0.120482899335375	0.904100625314942	1	WCA2 - GCA3	0.786390647046579	0.431638661213396	0.604294125698755	WCA2 - GCA3	1.3	0.193600969171221	0.774403876684883	WCA2 - GCA3	-1.13503744982291	0.256359627540954	0.512719255081908

Reaction center comparison	KO.K02704..psbB. Comparison	KO.K02704..psbB. Z	KO.K02704..psbB. P.unadj	KO.K02704..psbB. P.adj
1	RCA3 - RCA4	0.474879903960298	0.634872575947997	0.808019642115633
2	RCA3 - RCA5	2.65261449071734	0.00798710353918702	0.223638899097237
3	RCA4 - RCA5	2.30161346944969	0.0213569798629216	0.199331812053935
4	RCA3 - RCA6	0.435638176782363	0.663099267416823	0.807251282072654
5	RCA4 - RCA6	-0.0777204680682654	0.938050412188565	0.938050412188565
6	RCA5 - RCA6	-2.5868754037864	0.00968506061939602	0.135590848671544
7	RCA3 - BG1	-0.356159927970223	0.72172079967615	0.842007599622175
8	RCA4 - BG1	-0.654307567765093	0.512913671232713	0.718079139725798
9	RCA5 - BG1	-1.95390091428533	0.0507129364379418	0.354990555065593
10	RCA6 - BG1	-0.62789648972752	0.530071750688177	0.706762334250903
11	RCA3 - WCA1	0.660395349406222	0.509000155817716	0.750105492784002
12	RCA4 - WCA1	0.254452943019758	0.799145653892278	0.860618396499377
13	RCA5 - WCA1	-1.70818615189558	0.0876018072225749	0.3504072288903
14	RCA6 - WCA1	0.335235687049183	0.737447303605571	0.825940980038239
15	BG1 - WCA1	0.781660832781895	0.434413925669076	0.67575499548523
16	RCA3 - WCA2	1.89178707865113	0.0585193555905609	0.327708391307141
17	RCA4 - WCA2	1.49056872307218	0.136074761024114	0.346372118970472
18	RCA5 - WCA2	-0.85063090626265	0.39497441549305	0.6505460961062
19	RCA6 - WCA2	1.7041339945892	0.0883560569702831	0.309246199395991
20	BG1 - WCA2	1.49921956945164	0.13381667986806	0.374686703630568
21	WCA1 - WCA2	1.01364892530068	0.310750303270681	0.580067232771938
22	RCA3 - GCA3	1.87238445927348	0.061153433494391	0.285382689640491
23	RCA4 - GCA3	1.43629121100891	0.150919493921053	0.352145485815792
24	RCA5 - GCA3	-1.21969429834331	0.222580783821873	0.445161567643745
25	RCA6 - GCA3	1.69637408468484	0.089815058350788	0.279424625980229
26	BG1 - GCA3	1.42794115856893	0.153308795594074	0.330203559741082
27	WCA1 - GCA3	0.90406341640532	0.365961775557665	0.640433107225914
28	WCA2 - GCA3	-0.252022603556317	0.801023592474834	0.830691132936865

Table S3. Kruskal-Wallace significance for carbon fixation pathway genes.

	Comparison	Z	P.unadj	P.adj
**KO:K14468, malonyl-CoA reductase / 3-hydroxypropionate dehydrogenase, mcr.1**	RCA3 - RCA4	-0.941119812536102	0.346643463463169	0.647067798464582
**KO:K14468, malonyl-CoA reductase / 3-hydroxypropionate dehydrogenase, mcr.2**	RCA3 - RCA5	-0.458760872853754	0.646405895209873	0.786928915907672
**KO:K14468, malonyl-CoA reductase / 3-hydroxypropionate dehydrogenase, mcr.3**	RCA4 - RCA5	0.279053928209984	0.780203439108858	0.873827851801922
**KO:K14468, malonyl-CoA reductase / 3-hydroxypropionate dehydrogenase, mcr.4**	RCA3 - RCA6	-2.21386183185092	0.0268382850466698	0.250490660435585
**KO:K14468, malonyl-CoA reductase / 3-hydroxypropionate dehydrogenase, mcr.5**	RCA4 - RCA6	-1.64066455613526	0.100867066260627	0.470712975882927
**KO:K14468, malonyl-CoA reductase / 3-hydroxypropionate dehydrogenase, mcr.6**	RCA5 - RCA6	-1.48292440040885	0.138094497735195	0.552377990940779
**KO:K14468, malonyl-CoA reductase / 3-hydroxypropionate dehydrogenase, mcr.7**	RCA3 - BG1	-1.24606649271243	0.212739984971142	0.541519961744724
**KO:K14468, malonyl-CoA reductase / 3-hydroxypropionate dehydrogenase, mcr.8**	RCA4 - BG1	-0.567434861048148	0.57041877068099	0.798586278953386
**KO:K14468, malonyl-CoA reductase / 3-hydroxypropionate dehydrogenase, mcr.9**	RCA5 - BG1	-0.67943592328026	0.496861694774052	0.732217234403867
**KO:K14468, malonyl-CoA reductase / 3-hydroxypropionate dehydrogenase, mcr.10**	RCA6 - BG1	0.803571289334435	0.421644609843247	0.737878067225682
**KO:K14468, malonyl-CoA reductase / 3-hydroxypropionate dehydrogenase, mcr.11**	RCA3 - WCA1	0.325676469686203	0.744669148918406	0.868780673738141
**KO:K14468, malonyl-CoA reductase / 3-hydroxypropionate dehydrogenase, mcr.12**	RCA4 - WCA1	1.22012704679468	0.222416716926688	0.518972339495605
**KO:K14468, malonyl-CoA reductase / 3-hydroxypropionate dehydrogenase, mcr.13**	RCA5 - WCA1	0.722374253004292	0.470064412132969	0.731211307762397
**KO:K14468, malonyl-CoA reductase / 3-hydroxypropionate dehydrogenase, mcr.14**	RCA6 - WCA1	2.37531425173307	0.0175340150437555	0.245476210612577
**KO:K14468, malonyl-CoA reductase / 3-hydroxypropionate dehydrogenase, mcr.15**	BG1 - WCA1	1.4702309163422	0.141499224110424	0.440219808343542
**KO:K14468, malonyl-CoA reductase / 3-hydroxypropionate dehydrogenase, mcr.16**	RCA3 - WCA2	-0.734130348385798	0.462869298755216	0.76237296265565
**KO:K14468, malonyl-CoA reductase / 3-hydroxypropionate dehydrogenase, mcr.17**	RCA4 - WCA2	0.0849840633393807	0.932274060482491	0.932274060482491
**KO:K14468, malonyl-CoA reductase / 3-hydroxypropionate dehydrogenase, mcr.18**	RCA5 - WCA2	-0.185114036414673	0.853139592405966	0.918765714898733
**KO:K14468, malonyl-CoA reductase / 3-hydroxypropionate dehydrogenase, mcr.19**	RCA6 - WCA2	1.47973148346512	0.13894492007742	0.486307220270971
**KO:K14468, malonyl-CoA reductase / 3-hydroxypropionate dehydrogenase, mcr.20**	BG1 - WCA2	0.566393860323831	0.571126067520003	0.761501423360005
**KO:K14468, malonyl-CoA reductase / 3-hydroxypropionate dehydrogenase, mcr.21**	WCA1 - WCA2	-1.0053491020748	0.314728779103252	0.677877370376234
**KO:K14468, malonyl-CoA reductase / 3-hydroxypropionate dehydrogenase, mcr.22**	RCA3 - GCA3	0.550822632755244	0.581755267567572	0.740415795086001
**KO:K14468, malonyl-CoA reductase / 3-hydroxypropionate dehydrogenase, mcr.23**	RCA4 - GCA3	1.86492805661418	0.0621914891437908	0.348272339205228
**KO:K14468, malonyl-CoA reductase / 3-hydroxypropionate dehydrogenase, mcr.24**	RCA5 - GCA3	0.978280311063139	0.327935705038515	0.655871410077031
**KO:K14468, malonyl-CoA reductase / 3-hydroxypropionate dehydrogenase, mcr.25**	RCA6 - GCA3	3.13260700142661	0.00173261260959842	0.0485131530687557
**KO:K14468, malonyl-CoA reductase / 3-hydroxypropionate dehydrogenase, mcr.26**	BG1 - GCA3	1.91450059128265	0.0555562236536922	0.388893565575846
**KO:K14468, malonyl-CoA reductase / 3-hydroxypropionate dehydrogenase, mcr.27**	WCA1 - GCA3	0.12693868343982	0.898988929318103	0.932284815589144
**KO:K14468, malonyl-CoA reductase / 3-hydroxypropionate dehydrogenase, mcr.28**	WCA2 - GCA3	1.40695838195197	0.159439730025726	0.446431244072033
**KO:K00031, isocitrate dehydrogenase.1**	RCA3 - RCA4	1.79646220851159	0.0724210378804665	0.135185937376871
**KO:K00031, isocitrate dehydrogenase.2**	RCA3 - RCA5	8.9330317799087	4.14490547435009e-19	5.80286766409012e-18
**KO:K00031, isocitrate dehydrogenase.3**	RCA4 - RCA5	8.12334746580942	4.53498596579347e-16	4.23265356807391e-15
**KO:K00031, isocitrate dehydrogenase.4**	RCA3 - RCA6	3.17533251718929	0.00149664890306828	0.00380965175326472
**KO:K00031, isocitrate dehydrogenase.5**	RCA4 - RCA6	1.6282503260955	0.103471817599521	0.144860544639329
**KO:K00031, isocitrate dehydrogenase.6**	RCA5 - RCA6	-6.59778697021403	4.17340346459024e-11	1.94758828347545e-10
**KO:K00031, isocitrate dehydrogenase.7**	RCA3 - BG1	0.0126394984399264	0.989915407853104	0.989915407853104
**KO:K00031, isocitrate dehydrogenase.8**	RCA4 - BG1	-2.03380227817406	0.0419715347562694	0.0904002287058111
**KO:K00031, isocitrate dehydrogenase.9**	RCA5 - BG1	-9.71290812781784	2.65647521715104e-22	7.43813060802292e-21
**KO:K00031, isocitrate dehydrogenase.10**	RCA6 - BG1	-3.57051322769111	0.000356282460707256	0.00110843432220035
**KO:K00031, isocitrate dehydrogenase.11**	RCA3 - WCA1	1.08566357433205	0.277627861813471	0.337981744816399
**KO:K00031, isocitrate dehydrogenase.12**	RCA4 - WCA1	-0.57643652441588	0.564320162756104	0.658373523215455
**KO:K00031, isocitrate dehydrogenase.13**	RCA5 - WCA1	-7.88423612614996	3.16465377336219e-15	2.21525764135353e-14
**KO:K00031, isocitrate dehydrogenase.14**	RCA6 - WCA1	-1.98508157738604	0.0471353960999581	0.0942707921999161
**KO:K00031, isocitrate dehydrogenase.15**	BG1 - WCA1	1.18956496428061	0.234217423180966	0.29809490223032
**KO:K00031, isocitrate dehydrogenase.16**	RCA3 - WCA2	1.54633569831214	0.122023514759647	0.162698019679529
**KO:K00031, isocitrate dehydrogenase.17**	RCA4 - WCA2	-0.159498364656411	0.873276246640714	0.940451342536153
**KO:K00031, isocitrate dehydrogenase.18**	RCA5 - WCA2	-7.85300844285663	4.06174311954612e-15	2.27457614694583e-14
**KO:K00031, isocitrate dehydrogenase.19**	RCA6 - WCA2	-1.66699834510914	0.0955147339377967	0.140758555276753
**KO:K00031, isocitrate dehydrogenase.20**	BG1 - WCA2	1.7196726671884	0.0854919589152811	0.140810285272228
**KO:K00031, isocitrate dehydrogenase.21**	WCA1 - WCA2	0.401390531660051	0.688132619309043	0.770708533626128
**KO:K00031, isocitrate dehydrogenase.22**	RCA3 - GCA3	3.23042376598512	0.0012360686276273	0.00346099215735643
**KO:K00031, isocitrate dehydrogenase.23**	RCA4 - GCA3	1.69516248316287	0.0900446017376212	0.140069380480744
**KO:K00031, isocitrate dehydrogenase.24**	RCA5 - GCA3	-6.52241557625518	6.91840059203896e-11	2.76736023681559e-10
**KO:K00031, isocitrate dehydrogenase.25**	RCA6 - GCA3	0.0704289831132221	0.943852223330166	0.978809713083135
**KO:K00031, isocitrate dehydrogenase.26**	BG1 - GCA3	3.62965365825621	0.000283801751102928	0.000993306128860249
**KO:K00031, isocitrate dehydrogenase.27**	WCA1 - GCA3	2.04342498228763	0.0410103865155102	0.0956909018695238
**KO:K00031, isocitrate dehydrogenase.28**	WCA2 - GCA3	1.7295192794492	0.0837161990039705	0.146503348256948
**KO:K01601, RuBisCO large chain.1**	RCA3 - RCA4	0.356449109291752	0.721504257336869	0.841754966893014
**KO:K01601, RuBisCO large chain.2**	RCA3 - RCA5	2.63987427311068	0.00829367889607548	0.0387038348483522
**KO:K01601, RuBisCO large chain.3**	RCA4 - RCA5	2.94173702694612	0.00326376959618907	0.0228463871733235
**KO:K01601, RuBisCO large chain.4**	RCA3 - RCA6	-0.659910724262796	0.509311121735718	0.792261744922228
**KO:K01601, RuBisCO large chain.5**	RCA4 - RCA6	-1.32114262976361	0.186453811158764	0.4746097011314
**KO:K01601, RuBisCO large chain.6**	RCA5 - RCA6	-3.98760205525837	6.67444958067551e-05	0.00186884588258914
**KO:K01601, RuBisCO large chain.7**	RCA3 - BG1	-0.367355844038649	0.713353603871694	0.907904586745793
**KO:K01601, RuBisCO large chain.8**	RCA4 - BG1	-0.901098691787119	0.367535848436348	0.686066917081182
**KO:K01601, RuBisCO large chain.9**	RCA5 - BG1	-3.50256387551345	0.000460803281332374	0.00430083062576883
**KO:K01601, RuBisCO large chain.10**	RCA6 - BG1	0.332834012398016	0.739259582405293	0.827970732293928
**KO:K01601, RuBisCO large chain.11**	RCA3 - WCA1	1.08551612707636	0.277693124894969	0.598108269004549
**KO:K01601, RuBisCO large chain.12**	RCA4 - WCA1	0.970181160226803	0.331956199785017	0.663912399570033
**KO:K01601, RuBisCO large chain.13**	RCA5 - WCA1	-1.91810185380446	0.055098097465121	0.192843341127923
**KO:K01601, RuBisCO large chain.14**	RCA6 - WCA1	2.16176756607717	0.0306360966669291	0.122544386667716
**KO:K01601, RuBisCO large chain.15**	BG1 - WCA1	1.72929002323837	0.0837572013231467	0.260577959672012
**KO:K01601, RuBisCO large chain.16**	RCA3 - WCA2	0.0314938616257345	0.974875687431419	0.974875687431419
**KO:K01601, RuBisCO large chain.17**	RCA4 - WCA2	-0.358580954161841	0.719908596889252	0.87641046577822
**KO:K01601, RuBisCO large chain.18**	RCA5 - WCA2	-2.85987932782258	0.00423802237608758	0.0237329253060905
**KO:K01601, RuBisCO large chain.19**	RCA6 - WCA2	0.769003823141824	0.441891040942885	0.727820538023575
**KO:K01601, RuBisCO large chain.20**	BG1 - WCA2	0.440411921497183	0.659638792078691	0.923494308910167
**KO:K01601, RuBisCO large chain.21**	WCA1 - WCA2	-1.16272074079328	0.24494282369839	0.571533255296243
**KO:K01601, RuBisCO large chain.22**	RCA3 - GCA3	-0.0751829814419888	0.94006912486921	0.974886499864366
**KO:K01601, RuBisCO large chain.23**	RCA4 - GCA3	-0.640037968922747	0.522147915157659	0.769481138127076
**KO:K01601, RuBisCO large chain.24**	RCA5 - GCA3	-3.72839541516814	0.0001927028509338	0.0026978399130732
**KO:K01601, RuBisCO large chain.25**	RCA6 - GCA3	0.853041349875673	0.393636373978227	0.688863654461897
**KO:K01601, RuBisCO large chain.26**	BG1 - GCA3	0.419676222328066	0.674721997570975	0.899629330094633
**KO:K01601, RuBisCO large chain.27**	WCA1 - GCA3	-1.64787542744072	0.0993782367824008	0.278259062990722
**KO:K01601, RuBisCO large chain.28**	WCA2 - GCA3	-0.127358709541428	0.898656495066619	0.967783917764051
**KO:K15052, propionyl-CoA carboxylase.1**	RCA3 - RCA4	1.65449444494492	0.0980270910843206	0.274475855036098
**KO:K15052, propionyl-CoA carboxylase.2**	RCA3 - RCA5	4.17469532911321	2.98385110915919e-05	0.000835478310564574
**KO:K15052, propionyl-CoA carboxylase.3**	RCA4 - RCA5	2.95760325233174	0.00310040893702816	0.0217028625591971
**KO:K15052, propionyl-CoA carboxylase.4**	RCA3 - RCA6	1.6118945294349	0.106984892838734	0.272325181771323
**KO:K15052, propionyl-CoA carboxylase.5**	RCA4 - RCA6	0.149648647710987	0.881041824990383	0.913673003693731
**KO:K15052, propionyl-CoA carboxylase.6**	RCA5 - RCA6	-2.55468603782235	0.010628361639006	0.0595188251784336
**KO:K15052, propionyl-CoA carboxylase.7**	RCA3 - BG1	0.886880142191253	0.375143433915229	0.617883302919201
**KO:K15052, propionyl-CoA carboxylase.8**	RCA4 - BG1	-0.652757189107944	0.513912827090378	0.685217102787171
**KO:K15052, propionyl-CoA carboxylase.9**	RCA5 - BG1	-3.27255449092898	0.00106580325953523	0.0149212456334932
**KO:K15052, propionyl-CoA carboxylase.10**	RCA6 - BG1	-0.720487282161042	0.471225027339387	0.694436882394886
**KO:K15052, propionyl-CoA carboxylase.11**	RCA3 - WCA1	1.01379565941024	0.310680266921952	0.543690467113415
**KO:K15052, propionyl-CoA carboxylase.12**	RCA4 - WCA1	-0.469125625968256	0.638979842960236	0.745476483453609
**KO:K15052, propionyl-CoA carboxylase.13**	RCA5 - WCA1	-3.05435026696424	0.00225548573397564	0.0210512001837727
**KO:K15052, propionyl-CoA carboxylase.14**	RCA6 - WCA1	-0.555211330525389	0.578750119781452	0.704565363212202
**KO:K15052, propionyl-CoA carboxylase.15**	BG1 - WCA1	0.148348511939696	0.882067731876709	0.882067731876709
**KO:K15052, propionyl-CoA carboxylase.16**	RCA3 - WCA2	2.14713846437177	0.0317822598878445	0.111237909607456
**KO:K15052, propionyl-CoA carboxylase.17**	RCA4 - WCA2	0.66780362914745	0.504258949683851	0.705962529557392
**KO:K15052, propionyl-CoA carboxylase.18**	RCA5 - WCA2	-2.20434594301096	0.0275000242226204	0.110000096890481
**KO:K15052, propionyl-CoA carboxylase.19**	RCA6 - WCA2	0.455198021840224	0.648966783513997	0.726842797535677
**KO:K15052, propionyl-CoA carboxylase.20**	BG1 - WCA2	1.20851244181976	0.226850212980794	0.488600458727864
**KO:K15052, propionyl-CoA carboxylase.21**	WCA1 - WCA2	1.02315874034109	0.306232797452609	0.57163455524487
**KO:K15052, propionyl-CoA carboxylase.22**	RCA3 - GCA3	2.330916364318	0.0197577702164931	0.092202927676968
**KO:K15052, propionyl-CoA carboxylase.23**	RCA4 - GCA3	0.866573792407852	0.386175578199963	0.600717566088831
**KO:K15052, propionyl-CoA carboxylase.24**	RCA5 - GCA3	-2.03569977586955	0.0417805116220578	0.129983813935291
**KO:K15052, propionyl-CoA carboxylase.25**	RCA6 - GCA3	0.632124631079179	0.527305441400282	0.671116016327631
**KO:K15052, propionyl-CoA carboxylase.26**	BG1 - GCA3	1.38786130778362	0.165179281838501	0.385418324289836
**KO:K15052, propionyl-CoA carboxylase.27**	WCA1 - GCA3	1.19750576457088	0.231109482047302	0.462218964094604
**KO:K15052, propionyl-CoA carboxylase.28**	WCA2 - GCA3	0.183777899946228	0.8541876941182	0.9198944398196
**KO:K01602, RuBisCO small chain.1**	RCA3 - RCA4	0.158910431540932	0.873739441952744	0.906100162025068
**KO:K01602, RuBisCO small chain.2**	RCA3 - RCA5	1.55699788832305	0.119470986771701	0.477883947086803
**KO:K01602, RuBisCO small chain.3**	RCA4 - RCA5	1.74801474695025	0.0804614738801762	0.375486878107489
**KO:K01602, RuBisCO small chain.4**	RCA3 - RCA6	0.165607285106418	0.868466013779391	0.935271091762422
**KO:K01602, RuBisCO small chain.5**	RCA4 - RCA6	0.00428549564355496	0.996580679656862	0.996580679656862
**KO:K01602, RuBisCO small chain.6**	RCA5 - RCA6	-1.78619286079065	0.0740680366333199	0.414781005146591
**KO:K01602, RuBisCO small chain.7**	RCA3 - BG1	-0.752548979356139	0.451720986717485	0.63240938140448
**KO:K01602, RuBisCO small chain.8**	RCA4 - BG1	-1.08059093447834	0.279879116888221	0.522441018191345
**KO:K01602, RuBisCO small chain.9**	RCA5 - BG1	-2.30954686767918	0.0209132540827942	0.585571114318237
**KO:K01602, RuBisCO small chain.10**	RCA6 - BG1	-1.10897735562333	0.267439950770751	0.534879901541503
**KO:K01602, RuBisCO small chain.11**	RCA3 - WCA1	1.03377312244364	0.301242239508262	0.496163688601843
**KO:K01602, RuBisCO small chain.12**	RCA4 - WCA1	1.18594462200587	0.235644169741546	0.59982152297848
**KO:K01602, RuBisCO small chain.13**	RCA5 - WCA1	-0.764093177458341	0.44481171773707	0.655512005086208
**KO:K01602, RuBisCO small chain.14**	RCA6 - WCA1	1.22777914040028	0.219529888240544	0.614683687073524
**KO:K01602, RuBisCO small chain.15**	BG1 - WCA1	1.90274183406293	0.0570742403299591	0.799039364619428
**KO:K01602, RuBisCO small chain.16**	RCA3 - WCA2	-0.311399577664609	0.755496874928883	0.846156499920349
**KO:K01602, RuBisCO small chain.17**	RCA4 - WCA2	-0.540295467239169	0.58899328384327	0.749627815800525
**KO:K01602, RuBisCO small chain.18**	RCA5 - WCA2	-1.86839746598765	0.0617066900283127	0.575929106930919
**KO:K01602, RuBisCO small chain.19**	RCA6 - WCA2	-0.555967314285831	0.578233199443022	0.770977599257363
**KO:K01602, RuBisCO small chain.20**	BG1 - WCA2	0.441149401691529	0.659104841083198	0.802388502188241
**KO:K01602, RuBisCO small chain.21**	WCA1 - WCA2	-1.39334638242403	0.163515071079141	0.572302748776993
**KO:K01602, RuBisCO small chain.22**	RCA3 - GCA3	0.87981479532574	0.378959648276568	0.589492786207995
**KO:K01602, RuBisCO small chain.23**	RCA4 - GCA3	1.0742345172167	0.282717585603969	0.494755774806946
**KO:K01602, RuBisCO small chain.24**	RCA5 - GCA3	-1.14081248580515	0.253947963631803	0.592545248474208
**KO:K01602, RuBisCO small chain.25**	RCA6 - GCA3	1.13786134000231	0.255178386564072	0.549614986445694
**KO:K01602, RuBisCO small chain.26**	BG1 - GCA3	1.856451314539	0.063389256338695	0.443724794370865
**KO:K01602, RuBisCO small chain.27**	WCA1 - GCA3	-0.370680922756022	0.710875201087665	0.829354401268943
**KO:K01602, RuBisCO small chain.28**	WCA2 - GCA3	1.28394025155192	0.1991628619645	0.619617792778443
**KO:K08691, malyl-CoA/(S)-citramalyl-CoA lyase, mcl.1**	RCA3 - RCA4	-0.875660954240913	0.381214377895094	1
**KO:K08691, malyl-CoA/(S)-citramalyl-CoA lyase, mcl.2**	RCA3 - RCA5	-0.653197264742181	0.513629113393124	1
**KO:K08691, malyl-CoA/(S)-citramalyl-CoA lyase, mcl.3**	RCA4 - RCA5	-0.01992047682224	0.984106810243543	1
**KO:K08691, malyl-CoA/(S)-citramalyl-CoA lyase, mcl.4**	RCA3 - RCA6	-0.996528365411032	0.31899349202144	1
**KO:K08691, malyl-CoA/(S)-citramalyl-CoA lyase, mcl.5**	RCA4 - RCA6	-0.214179868038562	0.830406794793418	1
**KO:K08691, malyl-CoA/(S)-citramalyl-CoA lyase, mcl.6**	RCA5 - RCA6	-0.140859042454753	0.887981298849819	0.994539054711797
**KO:K08691, malyl-CoA/(S)-citramalyl-CoA lyase, mcl.7**	RCA3 - BG1	-1.10467979990469	0.269298359303348	1
**KO:K08691, malyl-CoA/(S)-citramalyl-CoA lyase, mcl.8**	RCA4 - BG1	-0.391037150294001	0.695769775798513	1
**KO:K08691, malyl-CoA/(S)-citramalyl-CoA lyase, mcl.9**	RCA5 - BG1	-0.285078658039919	0.775583880343026	1
**KO:K08691, malyl-CoA/(S)-citramalyl-CoA lyase, mcl.10**	RCA6 - BG1	-0.181015026749983	0.856355781214494	1
**KO:K08691, malyl-CoA/(S)-citramalyl-CoA lyase, mcl.11**	RCA3 - WCA1	-1.66647617959059	0.0956186087805806	1
**KO:K08691, malyl-CoA/(S)-citramalyl-CoA lyase, mcl.12**	RCA4 - WCA1	-0.864916648053908	0.387084539352515	1
**KO:K08691, malyl-CoA/(S)-citramalyl-CoA lyase, mcl.13**	RCA5 - WCA1	-0.606540317260596	0.544156018423112	1
**KO:K08691, malyl-CoA/(S)-citramalyl-CoA lyase, mcl.14**	RCA6 - WCA1	-0.574640444288113	0.565534493221279	1
**KO:K08691, malyl-CoA/(S)-citramalyl-CoA lyase, mcl.15**	BG1 - WCA1	-0.338530906422404	0.734963140531251	1
**KO:K08691, malyl-CoA/(S)-citramalyl-CoA lyase, mcl.16**	RCA3 - WCA2	-0.748331477354788	0.454260242566819	1
**KO:K08691, malyl-CoA/(S)-citramalyl-CoA lyase, mcl.17**	RCA4 - WCA2	-0.023002185311412	0.981648529785	1
**KO:K08691, malyl-CoA/(S)-citramalyl-CoA lyase, mcl.18**	RCA5 - WCA2	0	1	1
**KO:K08691, malyl-CoA/(S)-citramalyl-CoA lyase, mcl.19**	RCA6 - WCA2	0.159719141249985	0.873102321936738	1
**KO:K08691, malyl-CoA/(S)-citramalyl-CoA lyase, mcl.20**	BG1 - WCA2	0.318727629155838	0.749933061460078	1
**KO:K08691, malyl-CoA/(S)-citramalyl-CoA lyase, mcl.21**	WCA1 - WCA2	0.694879228972303	0.48713099068786	1
**KO:K08691, malyl-CoA/(S)-citramalyl-CoA lyase, mcl.22**	RCA3 - GCA3	-0.695238095238095	0.486906102086823	1
**KO:K08691, malyl-CoA/(S)-citramalyl-CoA lyase, mcl.23**	RCA4 - GCA3	0.221352738206059	0.824817789090383	1
**KO:K08691, malyl-CoA/(S)-citramalyl-CoA lyase, mcl.24**	RCA5 - GCA3	0.173880176985767	0.861959631776943	1
**KO:K08691, malyl-CoA/(S)-citramalyl-CoA lyase, mcl.25**	RCA6 - GCA3	0.417053005800816	0.676639637416194	1
**KO:K08691, malyl-CoA/(S)-citramalyl-CoA lyase, mcl.26**	BG1 - GCA3	0.579154125543267	0.562485181482578	1
**KO:K08691, malyl-CoA/(S)-citramalyl-CoA lyase, mcl.27**	WCA1 - GCA3	1.1047619047619	0.269262771543881	1
**KO:K08691, malyl-CoA/(S)-citramalyl-CoA lyase, mcl.28**	WCA2 - GCA3	0.202030508910442	0.839892873271269	1
**KO:K00855, phosphoribulokinase.1**	RCA3 - RCA4	0.043899588891707	0.964984443003393	0.964984443003393
**KO:K00855, phosphoribulokinase.2**	RCA3 - RCA5	2.9615992823517	0.00306045805975192	0.0171385651346108
**KO:K00855, phosphoribulokinase.3**	RCA4 - RCA5	3.34784848544793	0.000814415235241913	0.00760120886225785
**KO:K00855, phosphoribulokinase.4**	RCA3 - RCA6	0.105633385770669	0.915873234919607	1
**KO:K00855, phosphoribulokinase.5**	RCA4 - RCA6	0.0712840484923332	0.94317169038987	0.978103975219125
**KO:K00855, phosphoribulokinase.6**	RCA5 - RCA6	-3.27846568754565	0.00104373052307289	0.00730611366151021
**KO:K00855, phosphoribulokinase.7**	RCA3 - BG1	-0.322594778107901	0.747002148938952	0.871502507095444
**KO:K00855, phosphoribulokinase.8**	RCA4 - BG1	-0.419380467320466	0.674938096879319	0.821663770113954
**KO:K00855, phosphoribulokinase.9**	RCA5 - BG1	-3.67184058524436	0.000240809883370584	0.00674267673437636
**KO:K00855, phosphoribulokinase.10**	RCA6 - BG1	-0.488763265222749	0.625009307960282	0.79546639194945
**KO:K00855, phosphoribulokinase.11**	RCA3 - WCA1	1.28551609224396	0.198611997026004	0.327125642160477
**KO:K00855, phosphoribulokinase.12**	RCA4 - WCA1	1.41583263725134	0.156824511442631	0.292739088026245
**KO:K00855, phosphoribulokinase.13**	RCA5 - WCA1	-1.783534185839	0.0744993882320511	0.208598287049743
**KO:K00855, phosphoribulokinase.14**	RCA6 - WCA1	1.3486253918122	0.177457320578965	0.310550311013189
**KO:K00855, phosphoribulokinase.15**	BG1 - WCA1	1.77867441966506	0.0752931553530895	0.191655304535137
**KO:K00855, phosphoribulokinase.16**	RCA3 - WCA2	-0.790871311051719	0.429019091241545	0.600626727738162
**KO:K00855, phosphoribulokinase.17**	RCA4 - WCA2	-0.915730061400507	0.359808493859757	0.530244096214378
**KO:K00855, phosphoribulokinase.18**	RCA5 - WCA2	-3.65703422738894	0.000255150321218365	0.00357210449705711
**KO:K00855, phosphoribulokinase.19**	RCA6 - WCA2	-0.973933243269183	0.330089686425209	0.513472845550325
**KO:K00855, phosphoribulokinase.20**	BG1 - WCA2	-0.553762534084827	0.579741372145126	0.772988496193501
**KO:K00855, phosphoribulokinase.21**	WCA1 - WCA2	-2.0578489472534	0.0396046357571572	0.1232144223556
**KO:K00855, phosphoribulokinase.22**	RCA3 - GCA3	1.43179827396944	0.152201561397953	0.304403122795906
**KO:K00855, phosphoribulokinase.23**	RCA4 - GCA3	1.67608070744947	0.0937223851207115	0.21868556528166
**KO:K00855, phosphoribulokinase.24**	RCA5 - GCA3	-2.32143949085926	0.0202631362652211	0.0945613025710317
**KO:K00855, phosphoribulokinase.25**	RCA6 - GCA3	1.59138263688774	0.111523489473659	0.240204438866343
**KO:K00855, phosphoribulokinase.26**	BG1 - GCA3	2.10696687686966	0.0351204508316474	0.122921577910766
**KO:K00855, phosphoribulokinase.27**	WCA1 - GCA3	-0.0997788078932768	0.920519932927144	0.991329158536924
**KO:K00855, phosphoribulokinase.28**	WCA2 - GCA3	2.3035577566433	0.0212474814801773	0.084989925920709
**KO:K15231, ATP-citrate lyase beta-subunit.1**	RCA6 - BG1	-0.654653670707977	0.512690760261924	0.615228912314308
**KO:K15231, ATP-citrate lyase beta-subunit.2**	RCA6 - WCA2	0	1	1
**KO:K15231, ATP-citrate lyase beta-subunit.3**	BG1 - WCA2	0.755928946018454	0.449691797968891	0.674537696953336
**KO:K15231, ATP-citrate lyase beta-subunit.4**	RCA6 - GCA3	-1.60356745147455	0.108809430040546	0.326428290121637
**KO:K15231, ATP-citrate lyase beta-subunit.5**	BG1 - GCA3	-0.801783725737273	0.422678074170635	0.845356148341271
**KO:K15231, ATP-citrate lyase beta-subunit.6**	WCA2 - GCA3	-2.02837021134844	0.0425224779980516	0.25513486798831
**KO:K15230, ATP-citrate lyase alpha-subunit.1**	BG1 - WCA1	0.288675134594813	0.772829992684448	0.772829992684448
**KO:K15230, ATP-citrate lyase alpha-subunit.2**	BG1 - WCA2	-0.942809041582063	0.34577858615116	0.51866787922674
**KO:K15230, ATP-citrate lyase alpha-subunit.3**	WCA1 - WCA2	-1.29636243217534	0.194850648608869	0.389701297217739
**KO:K15230, ATP-citrate lyase alpha-subunit.4**	BG1 - GCA3	-1.53206469257085	0.125506471437469	0.376519414312407
**KO:K15230, ATP-citrate lyase alpha-subunit.5**	WCA1 - GCA3	-1.88561808316413	0.0593464387919199	0.356078632751519
**KO:K15230, ATP-citrate lyase alpha-subunit.6**	WCA2 - GCA3	-0.833333333333333	0.404656761927286	0.485588114312743

Supplemental references

1. Gaby, J.C. & Buckley D.H. (2014) A comprehensive aligned nifH gene database: A multipurpose tool for studies of nitrogen-fixing bacteria. Database. 2014:1–8.

2. Imhoff, J.F. *et al* (2018) Photosynthesis is widely distributed among Proteobacteria as demonstrated by the phylogeny of PufLM reaction center proteins. Front Microbiol. 8(JAN):1–11.
